# Triple therapy in type 2 diabetes; a systematic review and network meta-analysis

**DOI:** 10.7717/peerj.1461

**Published:** 2015-12-07

**Authors:** Martin J. Downes, Emilie K. Bettington, Jenny E. Gunton, Erika Turkstra

**Affiliations:** 1 Centre for Applied Health Economics, Menzies Health Institute Queensland, Griffith University, Queensland, Australia; 2 Chair of Medicine, Westmead Hospital, University of Sydney, Westmead, New South Wales, Australia

**Keywords:** Oral antidiabetic drugs, Anti-diabetic medication, Network meta-analysis, Type 2 diabetes, Glycated haemoglobin

## Abstract

**Aims.** The purpose was to evaluate the evidence for triple therapy regimen using medicines available in Australia for type 2 diabetes.

**Methods.** A systematic literature review was performed to update the relevant evidence from 2002 to 2014 on triple therapy for type 2 diabetes. A multiple-treatments network meta-analysis was undertaken to summarise the comparative efficacy and harms of different triple therapies.

**Results.** Twenty seven trials were identified, most were six months of duration. The following combinations were included in the network meta-analysis: metformin (MET) + sulfonylureas (SU) (used as reference combination); MET + SU+ dipeptidyl peptidase 4 inhibitors (DPP-4-i); MET + SU+ thiazolidinediones (TZD); MET + SU+ glucagon-like peptide-1 receptor agonists (GLP-1-RA); MET + SU+ insulins; MET + TZD + DPP-4-i; and MET + SU+ sodium/glucose cotransporter 2 inhibitors (SGLT2-i). For HbA_1c_ reduction, all triple therapies were statistically superior to MET+SU dual therapy, except for MET + TZD + DPP-4-i. None of the triple therapy combinations demonstrated differences in HbA_1c_ compared with other triple therapies. MET + SU + SGLT2-i and MET + SU + GLP-1-RA resulted in significantly lower body weight than MET + SU + DPP-4-i, MET+SU+insulin and MET + SU + TZDs; MET + SU + DPP-4-i resulted in significantly lower body weight than MET + SU + insulin and MET + SU + TZD. MET + SU + insulin, MET + SU + TZD and MET + SU + DPP-4-i increased the odds of hypoglycaemia when compared to MET + SU. MET + SU + GLP-1-RA reduced the odds of hypoglycaemia compared to MET + SU + insulin.

**Conclusion.** Care when choosing a triple therapy combination is needed as there is often a risk of increased hypoglycaemia events associated with this regimen and there are very limited data surrounding the long-term effectiveness and safety of combined therapies.

## Introduction

Type 2 diabetes is a major health concern worldwide and has an increasing prevalence and impact on health services with an estimated worldwide change in prevalence from 153 million in 1980 to 347 million in 2008 (Danaei et al., 2011) and 1.5 million deaths due to type 2 diabetes in 2012 (WHO, 2013).

Type 2 diabetes is a relatively difficult disease to manage with most international clinical guidelines recommending an individualised approach to the management of type 2 diabetes and an optimal HbA_1*c*_ target with regards to each patient (American Diabetes Association, 2014; Inzucchi et al., 2015; Canadian Agency for Drugs and Technologies in Health , 2013; Gunton et al., 2014; National Institute for Health and Clinical Excellence, 2011; New Zealand Guidelines Group, 2011). The balance for treatment is between optimal management of the disease and the prevention of microvascular events, and severe hypoglycaemia. Other important considerations are cost, efficacy, potential side effects, effects on body weight, comorbidities, and patient preferences and abilities which are critical for compliance and management of therapeutic strategies (e.g., oral or injectable medications).

The consensus between the different guidelines is that metformin is considered the first line of pharmacotherapy unless there are contraindications or patient intolerance (American Diabetes Association, 2014; Gunton et al., 2014; National Institute for Health and Clinical Excellence, 2011; New Zealand Guidelines Group, 2011). If either of these is present, sulfonylureas (SU) are often considered the most appropriate alternative to metformin (MET) (Gunton et al., 2014; National Institute for Health and Clinical Excellence, 2011; New Zealand Guidelines Group, 2011). International guidelines advise that if treatment with monotherapy does not result in optimal blood glucose levels then dual therapy should be initiated (American Diabetes Association, 2014; Inzucchi et al., 2015; Canadian Agency for Drugs and Technologies in Health , 2013; Gunton et al., 2014; National Institute for Health and Clinical Excellence, 2011; New Zealand Guidelines Group, 2011). NICE, Canada, Australia and New Zealand consider that MET and SU is the recommended dual therapy combination, unless contraindicated for the individual patient (American Diabetes Association, 2014; Inzucchi et al., 2015; Canadian Agency for Drugs and Technologies in Health , 2013; Gunton et al., 2014; National Institute for Health and Clinical Excellence, 2011; New Zealand Guidelines Group, 2011). A consensus from the American Diabetes Association (ADA) and the European Association for the Study of Diabetes (EASD) recommends trying a different first line to metformin and then a combination of drug for add on therapy (Inzucchi et al., 2015). In this scenario, other oral medications such as dipeptidyl peptidase-4 inhibitors (DPP-4-i) and thiazoldinediones (TZD) are generally recommended. If dual therapy is ineffective in controlling blood glucose, a third agent can be used to assist treatment. Given the number of medications available for type 2 diabetes; clinicians and patients need information about their effectiveness and safety to make informed choices.

The objective of this review was to summarize the benefits and harms of medications in triple therapy combination, for the treatment of adults with type 2 diabetes. This review includes those medications available in Australia in 2014, i.e., MET, SU, TZD, DPP-4-i, glucagon-like peptide-1 receptor agonists (GLP-1-RA), insulins, and sodium glucose co-transporter 2 inhibitors (SGLT2-i). The outcomes of interest were change in glycated haemoglobin, change in body weight and odds of hypoglycaemia events.

Our hypotheses are:

(1) Triple therapies for type 2 diabetes patients who are insufficiently managed with dual therapies have superior efficacy and inferior safety compared to dual therapy after six months of treatment and
(2) Triple therapies for type 2 diabetes have equal glycaemic efficacy and differing safety profiles.

## Methods

### Systematic literature review

The Cochrane Handbook for Systematic Reviews of Interventions (Higgins & Green, 2011) and the Preferred Reporting Items for Systematic Reviews and Meta-Analyses (PRISMA) reporting guidelines (Moher et al., 2009) were used in the development, execution and reporting of this review. A review protocol was not registered; however, the review was an update to a predetermined report on the Comparative Safety and Effectiveness of Type 2 Diabetes Medicines (Australian Government DoH, 2013).

The PICO for the systematic review was as follows:

Population: All patients with type 2 diabetes

Intervention: Any triple therapy combination for treatment of type 2 diabetes

Comparator: metformin plus sulphonylurea dual therapy, and other triple therapy combinations.

Outcome: HbA1c, body weight, hypoglycaemia and adverse events

The systematic literature review was performed in three stages: (1) identify the most relevant systematic literature reviews on the efficacy and safety of medications for glycaemic control; (2) update the literature search using the relevant systematic literature reviews as identified in step 1 as a starting point; and (3) identify the relevant randomized controlled trials (RCTs) from steps 1 and 2. Ovid MEDLINE and The Cochrane Library Database were used for searching the literature.

### Stage 1: identifying systematic reviews

Each bibliographic database was systematically searched using search terms for type 2 diabetes medications included in Table 1 on 5th March 2014. The key inclusion criteria were systematic reviews, English language publications, patients with type 2 diabetes, reviews published since 2010, reviews that include relevant outcomes, i.e., change in body weight, frequency of hypoglycaemic events, and/or total number severe adverse events. The key exclusion criteria was reviews that reported only on short-term treatment outcomes (<24 weeks). Supplementary Document—Stage 1, contains full details of the searches and terms used in each database. The searches were carried out by MJD and the results extracted and imported into the bibliographical software Endnote X7 (Thomson Reuters, New York, NY, USA). Duplicates were removed, articles that did not meet the inclusion criteria or met the exclusion criteria were also removed. The remaining articles were checked independently by two staff members for inclusion. If two systematic reviews were published based on the same data, the most comprehensive version of the paper was included. The review which was the most recent, included most therapeutic groups and outcomes was preferred.

**Table 1:** List of medicine name and medicine groups listed used for identifying systematic reviews and RCTs of diabetes medication in November 2014.

Medicine group	Medicine names
Biguanide	Metformin
Sulfonylureas	Gliclazide, Glimepiride, Glipizide, Glibenclamide
Thiazolidinedione	Pioglitazone, Rosiglitazone
Alpha-glucosidase inhibitors	Acarbose
Dipeptidyl peptidase-4 inhibitors	Alogliptin, Sitagliptin, Saxagliptin, Linagliptin, Vildagliptin
Glucagon-like peptide-1 receptor agonists	Exenatide, Liraglutide
Insulins	Aspart, Lispro, Glulisine, Neutral, Detemir, Glargine, Isophane
Sodium glucose co-transporter 2 inhibitors	Canagliflozin, Dapagliflozin

Bennett et al. (2011) was considered the most relevant systematic literature review. Bennett et al. (2011) did not include SGLT2-i, DPP-4-i or insulin. Therefore for the SGLT2-i and DPP-4-i two further systematic reviews were included; Berhan & Barker (2013) (SGLT2-i), and Monami et al. (2010) (DPP-4-i). We did not identify a systematic review on insulin in triple therapy that matched the inclusion and exclusion criteria. The clinical trials from the systematic literature reviews were extracted for further consideration.

### Stage 2: updating identified systematic reviews

Database searches were carried out to update the three identified systematic reviews in November 2014. Each bibliographic database was systematically searched using different search terms for each search strategy. Supplementary Document—Stage 2 contains full details of each search and the terms used in each database. The flow chart of the selection process is presented in Fig. S1.

**Figure 1:**
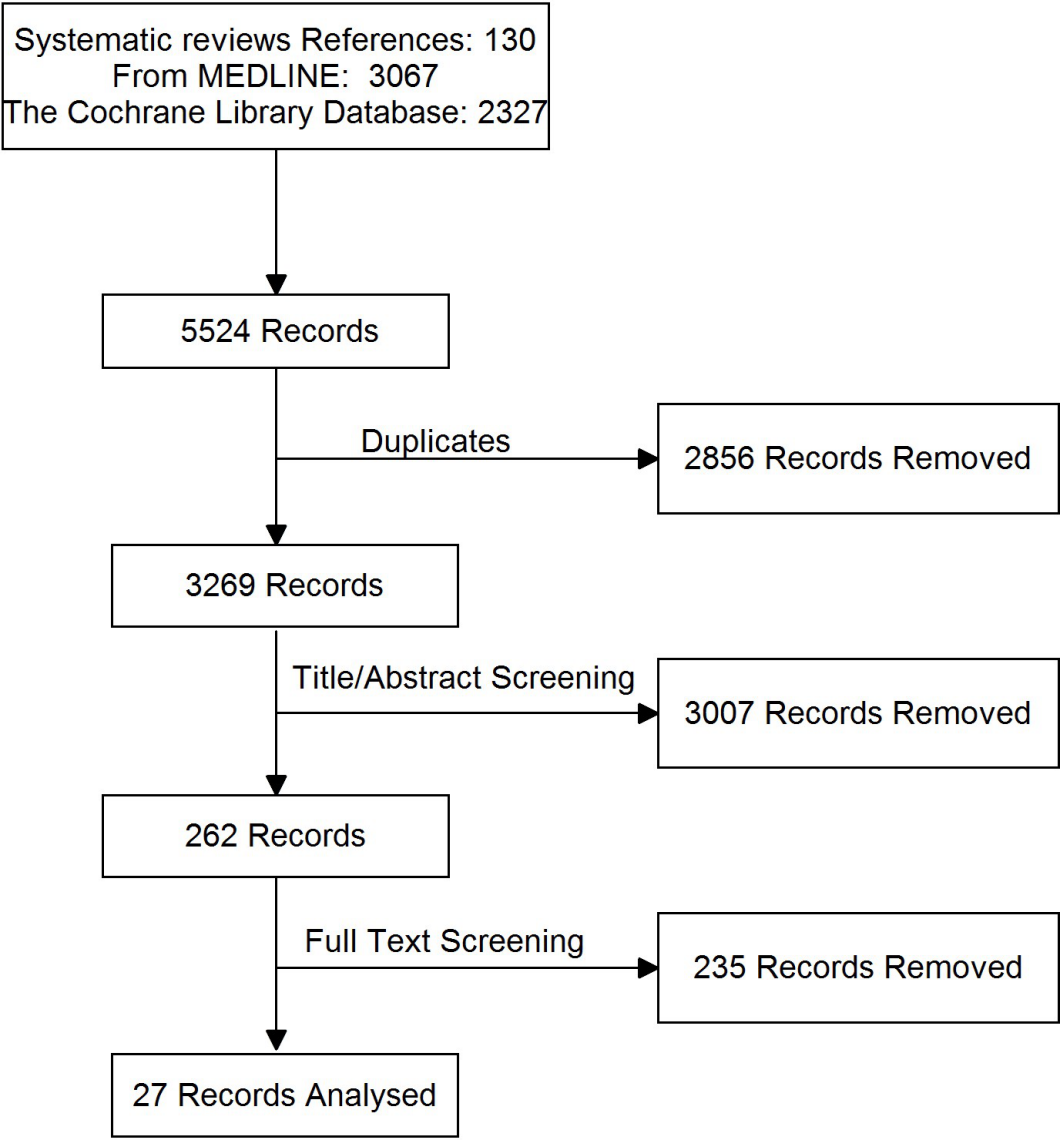
PRISMA flow diagram for RCTs in a systematic review of triple therapy in type 2 diabetes. Flow diagram showing the total number of records identified and the number of records filtered at each stage of the selection process from the systematic search for randomised control trials of type 2 diabetes in November 2014.

### Stage 3: identifying relevant RCTs

The articles identified through Stage 1 (included in relevant systematic literature reviews) and 2 (updated searches) were title checked by one author MJD and then independently by two authors (MJD and ET) and included in the final analysis if they met all of the following inclusion criteria: randomised controlled trial (RCT), English language publication, patients with type 2 diabetes, at least 100 participants in the trial, duration of treatment at least 24 weeks, published after 2002, trials that include any of the following relevant outcomes: HbA_1*c*_, body weight change, frequency of hypoglycaemic events, or frequency of other (serious) adverse events. Publications were excluded if they were not randomised control trials or if they were post hoc analysis of randomised control trials. If two publications used the same data, the most comprehensive version of the paper was included. The flow chart of the selection process is presented in Fig. 1.

#### Data extraction

Intention to treat data was extracted, where possible, from the included papers into a template in Excel. Extraction was performed by four different reviewers; for each publication, one reviewer extracted the data and one reviewer checked the extracted data for consistency. Risk of bias assessments were performed by one reviewer, and verified by a second reviewer. Risk of bias for the included RCTs was assessed using the Cochrane Collaboration’s ‘Risk of bias’ tool (Version 5.1.0.) (Higgins & Altman, 2011). Where any disagreements arose between the first reviewers extraction or bias assessment and the second reviewer, these were marked by the second reviewer and assessed by a third reviewer.

The following clinical outcomes were extracted at approximately six months after the start of the RCT: change in HbA_1*c*_, change in body weight, adverse events, hypoglycaemia (all, serious), and serious adverse events.

### Statistical analysis

Our primary outcome was mean difference in change in HbA_1*c*_ at approximately six months. To test the hypothesis that all triple therapies were superior to dual therapy, we considered the minimal clinically important difference (MCID) to be −0.3% (3.3 mmol/mol) as used by the Federal Drug Agency (FDA) (CDER, 2008) and the European Medicines Association (CHMP, 2011) and also quoted in Australia (Australian Government DoH, 2010). HbA_1*c*_ assays suffer from some variability, and a smaller MCID would be within that variability in an individual (Cohen, Haggerty & Herman, 2010). To demonstrate that triple therapies had similar efficacy the lower and the upper boundary of the confidence intervals needed to be within 0.3% (3.3 mmol/mol) using the FDA and the European Medicines Agency’s guidelines (CDER, 2008; CHMP, 2011).

Secondary outcomes were the difference in mean change in body weight (Kg) and difference in the hypoglycaemia odds ratio within six months. For those outcomes, we did not identify any MCIDs. Other outcomes investigated but not reported included adverse events, serious adverse events, severe hypoglycaemia and mortality.

Excel was used to consolidate and standardise the outcome measures and measures of variability; standard deviations, standard errors and 95% confidence intervals were imputed where necessary (Follmann et al., 1992). The data was then imported into STATA for meta-analysis and network meta-analysis. Where multiple trials were available head-to-head meta-analyses were performed using a random effects model.

A multiple-treatments network meta-analysis was undertaken to summarise the results of triple therapy for each of the outcomes where common treatment arms existed (HbA_1*c*_, body weight, and hypoglycaemia) using the trial data in the clinical evidence base. Different medications from the same class combinations were pooled, using the assumption that all these medications would have similar efficacy and safety. The network meta-analysis was conducted using STATA network package and mvmeta (Higgins et al., 2012; White, 2011) (The STATA .do file for HbA_1*c*_ is presented in Supplementary Document—Statistical Analyses). The network meta-analysis allowed for heterogeneity between studies during calculation (random effects). An inconsistency model was also applied to test for disagreement between direct and indirect evidence (Higgins et al., 2012). Possible covariates (Baseline HbA_1*c*_, age and Body Mass Index) were examined prior to carrying out the network analysis to ensure similarities in baseline characteristics. The measurements of treatment effect calculated were mean differences and their 95% confidence intervals (CI) for continuous data, and odds ratios and their 95% CI for dichotomous outcomes. Differences between treatments were considered statistically significant if there were no overlap in 95% CI. Some trials only compared treatments from the same combination group (e.g., MET+ SU+ INS vs MET+ SU+ INS). These trials were excluded from the network meta-analyses as they were considered to compare the same treatment and there did not randomise two different treatment regimens.

## Results

### Systematic literature review

The literature search identified 27 publications covering 26 trials with triple therapy; Fig. S2 provides a network for the direct comparisons identified in the search. Nine trials (10 publications) were excluded from the network meta-analyses as they were comparisons of the different drugs from the same treatment group e.g.,: MET+ SU+ INS vs. MET+ SU+ INS. One trial was excluded as it did not have a common comparator arm (Table S1). The majority of the remaining trials were of 24–26 weeks (11/17 trials) (Bergenstal et al., 2009; Dailey et al., 2004; DeFronzo et al., 2012; Heine et al., 2005; Hermansen et al., 2007; Liu et al., 2013; Lukashevich et al., 2014; Moses et al., 2014; Owens et al., 2011; Rosenstock et al., 2006; Russell-Jones et al., 2009), with only five trials longer than or equal to one year (Bosi et al., 2011; Derosa et al., 2013; Nauck et al., 2007; Schernthaner et al., 2013; Wilding et al., 2013). The included trials and comparisons are presented in Table 2.

**Figure 2:**
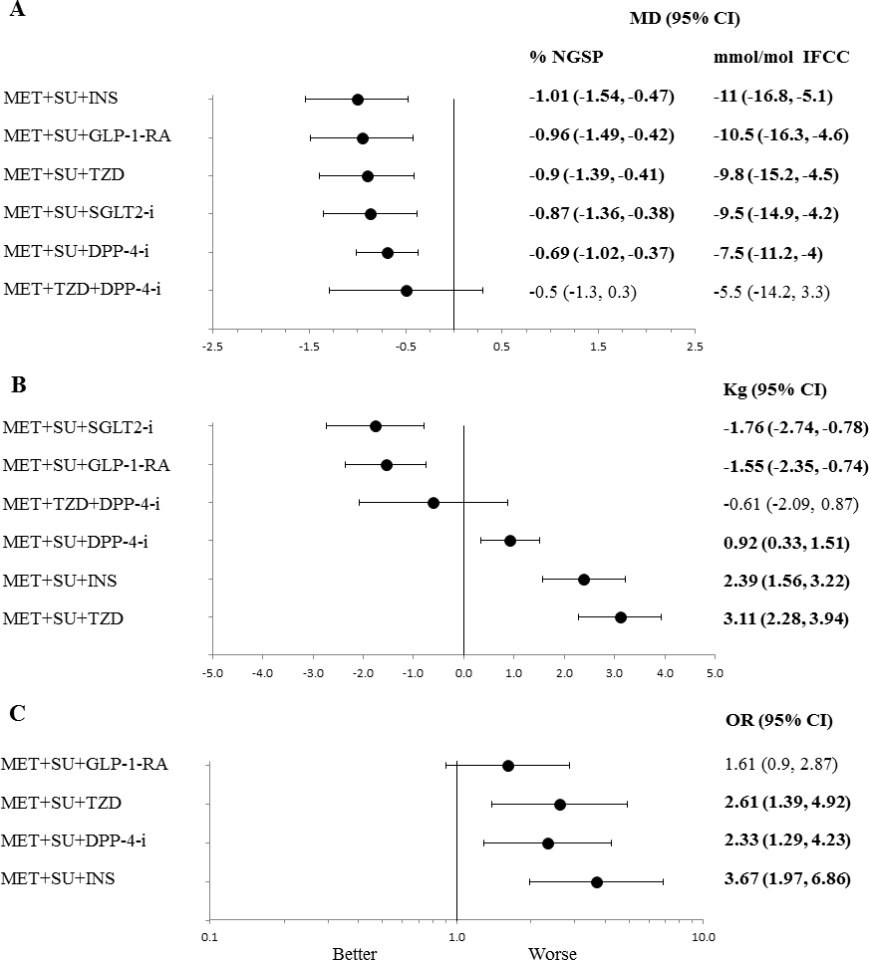
Line plots for different in efficacy and safety outcomes of triple therapy combinations compared to MET + SU dual therapy in type 2 diabetes. Line (forest) plots of mean difference of change in HbA1c (A), change in body weight (B), and hypoglycaemia (C), for different triple therapy combinations compared to MET + SU dual therapy. Abbreviations: CI, confidence interval; MD, mean difference; DPP-4-i, dipeptidyl peptidase-4 inhibitor; GLP-1-RA, glucagon-like peptide-1 receptor agonist; NGSP, National Glycohemoglobin Standardization Program; IFCC, International Federation of Clinical Chemistry and Laboratory Medicine. HbA1c, glycated haemoglobin; INS, insulin; MET, metformin; PBO, placebo; SU, sulfonylurea; TZD, thiazolidinedione; sodium/glucose cotransporter 2 inhibitors (SGLT2-i).

**Table 2:** Comparisons included in trials with triple therapy.

Intervention	Trials	Duration^c^	N	HbA_1*c*_	BW	AE	SAE	HypoG	Definition of hypoglycaemia
MET+ SU vs MET+ SU+ DPP-4-i	Hermansen et al. (2007) ^a^	24	441	x	x	x	x	x	ND
	Owens et al. (2011)	24	1,055	x	x	x	x	x	ND
	Lukashevich et al. (2014)	24	318	x	x	x	x	x	Symptoms suggestive of hypoglycaemia and a self-monitored plasma glucose measurement <3.1 mmol/l
MET + SU + TZD	Moses et al. (2014)	24	257	x	x	x	x	x	Symptomatic hypoglycaemia ± glucose measurement
	Dailey et al. (2004)	24	365	x	x	x		x	Symptomatic episodes with an associated fingerstick blood glucose ≤ 50 mg/dL
MET + SU + GLP-1-RA	Kendall et al. (2005)	30	733	x	x	x	x	x	Symptoms consistent with hypoglycaemia ± a documented plasma glucose concentration of <3.33 mmol/l
MET+ SU+ SGLT2-i	Russell-Jones et al. (2009) ^b^	26	581	x	x	x	x	x	Plasma glucose of <56 mg/dL (3.1 mmol/l)
	Wilding et al. (2013)	52	469	x	x	x	x	x	Symptomatic episodes with an associated fingerstick or plasma glucose ≤ 3.9 mmol/l
MET+ SU+ INS	Russell-Jones et al. (2009) ^b^	26	581	x	x	x	x	x	Plasma glucose of <56 mg/dL (3.1 mmol/l)
SU+ DPP-4-i	^a^ Hermansen et al. (2007)	24	441	x	x	x	x	x	ND
MET+ SU+ INS vs MET+ SU+ GLP-1-RA	Russell-Jones et al. (2009) ^b^	26	581	x	x	x	x	x	Plasma glucose of <56 mg/dL (3.1 mmol/L)
	Bergenstal et al. (2009)	24	372	x	x	x	x	x	Plasma glucose of <56 mg/dL (3.1 mmol/L)
	Heine et al. (2005)	26	549	x	x	x		x	Plasma glucose of <56 mg/dL (3.1 mmol/L)
	Nauck et al. (2007)	52	501	x	x	x	x	x	Any time a patient experienced a sign or symptom of hypoglycaemia or noted a blood glucose level <60 mg/dL (3.4 mmol/L).
MET+ SU+ TZD	Rosenstock et al. (2006)	24	216	x	x	x	x	x	Event with clinical symptoms consistent with hypoglycaemia, confirmed with a meter reading.
MET+ TZD+ DPP-4-i vs									
MET+ TZD	Bosi et al. (2011)	52	803	x	x	x	x	x	Blood glucose of <3.33 mmol/L with symptoms, or <2.78 mmol/L regardless of symptoms
	DeFronzo et al. (2012)	26	1,554	x		x	x	x	
MET+ SU+ TZD	Derosa et al. (2013)	52	453	x	x				NR
MET+ SU+ DPP-4-i vs MET+ SU+ SGLT2-i	Schernthaner et al. (2013)	52	755	x	x	x	x	x	Symptomatic episodes with fingerstick glucose ≤ 3.9 mmol/l
MET+ SU+ TZD	Liu et al. (2013)	24	119	x	x	x	x	x	ND

**Notes.**

AE adverse event BW body weight DPP-4-i dipeptidyl peptidase-4 inhibitor GLP-1-RA glucagon-like peptide-1 receptor agonist HbA_1*c*_ glycated haemoglobin HypoG hypoglycaemic event INS insulin MET metformin ND not defined NR not reported SAE serious adverse event SGLT2-i sodium glucose co-transporter 2 inhibitor SU sulfonylurea TZD thiazolidinedione

a One trial included three treatment arms (MET+ SU+ DPP4-i, MET+ SU and SU+ DPP4-i) and provided information for those comparisons.

b One trial included three treatment arms (MET+ SU+ GLP-1-RA, MET+ SU and MET+ SU+ INS) and provided information for those comparisons.

c Duration in months.

Of the 17 trials included in the network analysis, three trials were identified as having a high risk of bias (Dailey et al., 2004; Rosenstock et al., 2006; Kendall et al., 2005), nine trials as having an unclear risk of bias (Bergenstal et al., 2009; DeFronzo et al., 2012; Heine et al., 2005; Liu et al., 2013; Lukashevich et al., 2014; Owens et al., 2011; Nauck et al., 2007; Schernthaner et al., 2013; Wilding et al., 2013), and five trials as having a low risk of bias (Hermansen et al., 2007; Moses et al., 2014; Russell-Jones et al., 2009; Derosa et al., 2013) (Table S2). Only two of the studies were not funded by industry (Liu et al., 2013; Derosa et al., 2013).

Overall, the key features of the triple therapy trials varied (see Tables S3 and S4), which may limit the comparability of these trials. When reported, the trials recruited patients between 2002 and 2011 and most of the trials were performed in an international setting. The key features were that patients were adult patients with HbA_1*c*_ of 7% (53 mmol/mol) or higher. The primary outcome of interest for each study was mean difference in HbA_1*C*_ from the control group and the trials were powered to assess this outcome, although Rosenstock et al. (2006) did not provide a power calculation.

#### Baseline characteristics

Overall the baseline characteristics across the triple therapy trials were similar; however, there was some variation that may lead to heterogeneity between the trials and treatments within the network meta-analyses. Of those, the following potential effect modifiers varied between trial arms; baseline HbA_1*c*_ varied from 8.1% to 10.3%, duration of diabetes varied from 5 to 10 years and body mass index varied from 27 to 35 kg/m^2^ (Table S4).

The definition of hypoglycaemia, a secondary outcome in most of the trials, was variable and the differing definitions are presented in Table 2.

**Table 3:** Mean difference for change in HbA_1*c*_, weight and odds ratio of hypoglycaemia for dual therapy compared to triple therapy combinations in the network meta-analyses.

	HbA_1*c*_% NGSP (95% CI)		Weight Kg (95% CI)		Hypoglycaemia OR (95% CI)	
	Direct comparison^a^	Network	Direct comparison^a^	Network	Direct comparison^a^	Network
**vs MET**+**SU**						
MET+ SU+ SGLT2-i	**−0.82 (−0.83, −0.81)** (Wilding et al., 2013)	**−0.87 (−1.36, −0.38)**	**−1.4 (−1.45, −1.36)** (Wilding et al., 2013)	**−1.76 (−2.74, −0.78)**		–
MET+ SU+ GLP-1-RA	**−0.96 (−1.15, −0.77)** (Russell-Jones et al., 2009; Kendall et al., 2005)	**−0.96 (−1.49, −0.42)**	**−1.04 (−1.71, −0.37)** (Russell-Jones et al., 2009; Kendall et al., 2005)	**−1.55 (−2.35, −0.74)**	**1.7 (1.01, 2.86)** (Russell-Jones et al., 2009; Kendall et al., 2005)	1.61 (0.9, 2.87)
MET+ TZD+ DPP-4-i		−0.5 (−1.3, 0.3)		−0.61 (−2.09, 0.87)		–
MET+ SU+ DPP-4-i	**−0.71 (−0.79, −0.63)** (Hermansen et al., 2007; Lukashevich et al., 2014; Moses et al., 2014; Owens et al., 2011)	**0.69 (−1.02, −0.37)**	**0.71 (0.4, 1.01)** (Hermansen et al., 2007; Lukashevich et al., 2014; Moses et al., 2014; Owens et al., 2011)	**0.92 (0.33, 1.51)**	**2.39 (1.17, 4.88)** (Hermansen et al., 2007; Lukashevich et al., 2014; Moses et al., 2014; Owens et al., 2011)	**2.33 (1.29, 4.23)**
MET+ SU+ INS	**−0.85 (−1.13, −0.57)** (Russell-Jones et al., 2009)	**−1.01 (−1.54, −0.47)**	**2.02 (1.94, 2.1)** (Russell-Jones et al., 2009)	**2.39 (1.56, 3.22)**	**2.07 (1.17, 3.65)** (Russell-Jones et al., 2009)	**3.67 (1.97, 6.86)**
MET+ SU+ TZD	**−1 (−1.28, −0.72)** (Dailey et al., 2004)	**−0.9 (−1.39, −0.41)**	**2.97 (2.92, 3.02)** (Dailey et al., 2004)	**3.11 (2.28, 3.94)**	**3.41 (2.19, 5.32)** (Dailey et al., 2004)	**2.61 (1.39, 4.92)**
**vs SU**+**DPP-4-i**						
MET+ SU+ SGLT2-i		−0.31 (−1.1, 0.49)		**−2.89 (−4.20, −1.59)**		
MET+ SU+ GLP-1-RA		−0.4 (−1.18, 0.39)		**−2.60 (−3.79, −1.42)**		1.29 (0.31, 5.32)
MET+ TZD+ DPP-4-i		0.04 (−0.93, 1)		−1.59 (−3.32, 0.13)		
MET+ SU+ DPP-4-i	**−0.29(−0.31, −0.27)** (Hermansen et al., 2007)	−0.14 (−0.84, 0.57)	**−0.1(−0.11, −0.09)** (Hermansen et al., 2007)	−0.25 (−1.25, 0.75)	**2.42 (1.01, 5.8)** (Hermansen et al., 2007)	1.88 (0.61, 5.81)
MET+ SU+ INS		−0.43 (−1.22, 0.36)		**1.34 (0.14, 2.54)**		2.96 (0.71, 12.26)
MET+ SU+ TZD		−0.36 (−1.13, 0.41)		**2.03 (0.84, 3.23)**		2.1 (0.52, 8.56)
MET+ TZD		0.53 (−0.52, 1.58)		−1.81 (−3.94, 0.32)		
**vs MET**+**TZD**						
MET+ SU+ SGLT2-i		−0.62 (−1.48, 0.23)		**−1.83 (−3.36, −0.30)**		
MET+ SU+ GLP-1-RA		−0.72 (−1.53, 0.09)		**−1.59 (−2.95, −0.22)**		
MET+ TZD+ DPP-4-i	**−0.50 (−0.58, −0.41)** (DeFronzo et al., 2012; Bosi et al., 2011)	−0.41 (−0.92, 0.1)	**0.35 (0.33, 0.37)** (DeFronzo et al., 2012)	−0.2 (−1.33, 0.94)		
MET+ SU+ DPP-4-i		−0.46 (−1.24, 0.31)		0.86 (−0.44, 2.16)		
MET+ SU+ INS		−0.76 (−1.56, 0.05)		**2.36 (1.01, 3.72)**		
MET+ SU+ TZD		−0.71 (−1.42, −0.01)		**3.17 (1.92, 4.42)**		

**Notes.**

CI confidence interval DPP-4-i dipeptidyl peptidase-4 inhibitor GLP-1-RA glucagon-like peptide-1 receptor agonist HbA_1*c*_ glycated haemoglobin IFCC International Federation of Clinical Chemistry and Laboratory Medicine INS insulin MET metformin NGSP National Glycohemoglobin Standardization Program OR odds ratio SGLT2-i sodium glucose cotransporter 2 inhibitor SU sulfonylurea TZD thiazolidinedione **Bold** statistically significant

a Where multiple references were available a meta-analysis is presented.

**Table 4:** Mean difference for change in HbA_1*c*_, weight and odds ratio of hypoglycaemia for different triple medicine combinations compared to each other in the network meta-analyses.

	HbA_1*c*_ % NGSP (95% CI)		Weight Kg (95% CI)		Hypoglycaemia OR (95% CI)	
	Direct comparison^a^	Network	Direct comparison^a^	Network	Direct comparison^a^	Network
**MET**+**SU**+**SGLT2-i vs**						
MET+ SU+ GLP-1-RA		0.1 (−0.5, 0.7)		−0.66 (−1.88, 0.56)		–
MET+ TZD+ DPP-4-i		−0.3 (−1.1, 0.5)		**−1.84 (−3.3, -0.38)**		–
MET+ SU+ DPP-4-i	**−0.23 (−0.31, −0.15)** (Schernthaner et al., 2013)	−0.2 (−0.7, 0.3)	**−3 (−3.03, −2.97)** (Schernthaner et al., 2013)	**−2.63 (−3.6, −1.66)**		–
MET+ SU+ INS		0.1 (−0.5, 0.7)		**−3.68 (−4.95, −2.4)**		–
MET+ SU+ TZD		0.1 (−0.5, 0.6)		**−4.17 (−5.52, −2.82)**		–
**MET**+**SU**+**GLP-1-RA vs**						
MET+ TZD+ DPP-4-i		−0.4 (−1.1, 0.4)		**−1.6 (−2.89, −0.31)**		–
MET+ SU+ DPP-4-i		−0.3 (−0.7, 0.2)		**−2.34 (−3.25, −1.43)**		0.69 (0.3, 1.58)
MET+ SU+ INS	0 (−0.48, 0.48) (Bergenstal et al., 2009; Heine et al., 2005; Russell-Jones et al., 2009; Nauck et al., 2007)	0 (−0.3, 0.3)	**−4.11 (−4.76, −3.47)** (Bergenstal et al., 2009; Heine et al., 2005 Russell-Jones et al., 2009; Nauck et al., 2007)	**−3.78 (−4.43, −3.13)**	**0.42 (0.21, 0.86)** (Bergenstal et al., 2009; Russell-Jones et al., 2009)	**0.44 (0.25, 0.76)**
MET+ SU+ TZD		0 (−0.6, 0.6)		**−3.95 (−5.06, −2.85)**		0.62 (0.29, 1.31)
**MET**+**TZD**+**DPP-4-i vs**						
MET+ SU+ DPP-4-i		0.1 (−0.6, 0.8)		**−1.34 (−2.85, 0.17)**		-
MET+ SU+ INS		0.4 (−0.3, 1.2)		**−2.46 (−4.13, −0.79)**		-
MET+ SU+ TZD	**0.4 (0.33, 0.47)** (Derosa et al., 2013)	0.4 (−0.3, 1)	**−3.9 (−4.36, −3.44)** (Derosa et al., 2013)	**−3.22 (−4.88, −1.56)**		**-**
**MET**+**SU**+**DPP-4-i vs**						
MET+ SU+ INS		0.3 (−0.2, 0.8)		**−1.02 (−2, −0.04)**		0.63 (0.27, 1.48)
MET+ SU+ TZD	**0.23 (0.16, 0.31)** (Liu et al., 2013)	0.2 (−0.2, 0.7)	**−1.6 (−1.72, −1.49)** (Liu et al., 2013)	**−1.58 (−2.57, −0.6)**	0.82 (0.24, 2.84) (Liu et al., 2013)	0.89 (0.4, 2.01)
**MET**+**SU**+**INS vs**						
MET+ SU+ TZD	−0.15 (−0.55, 0.25) (Rosenstock et al., 2006)	−0.1 (−0.7, 0.5)	**−1.3 (−1.41, −1.19)** (Rosenstock et al., 2006)	−0.08 (−1.13, 0.97)	1.68 (0.98, 2.87) (Rosenstock et al., 2006)	1.41 (0.71, 2.8)

**Notes.**

CI confidence interval DPP-4-i dipeptidyl peptidase-4 inhibitor GLP-1-RA glucagon-like peptide-1 receptor agonist HbA_1*c*_ glycated haemoglobin IFCC International Federation of Clinical Chemistry and Laboratory Medicine INS insulin MET metformin NGSP National Glycohemoglobin Standardization Program OR odds ratio SGLT2-i sodium glucose cotransporter 2 inhibitor SU sulfonylurea TZD thiazolidinedione **Bold** statistically significant

a Where multiple references were available a meta-analysis is presented.

### Efficacy results

Two efficacy results were reported in the majority of the trials, change in HbA_1*c*_ and change in body weight. Seventeen RCTs were identified for inclusion in the network analysis for HbA_1*c*_ at six months (*N* = 9, 144) (Bergenstal et al., 2009; Bosi et al., 2011; Derosa et al., 2013; Nauck et al., 2007; Schernthaner et al., 2013; Wilding et al., 2013; Kendall et al., 2005) (Fig. S2). Sixteen RCTs were identified for inclusion in the network analysis for body weight change at six months (*N* = 8, 341) (Bergenstal et al., 2009; Dailey et al., 2004; DeFronzo et al., 2012; Heine et al., 2005; Hermansen et al., 2007; Liu et al., 2013; Lukashevich et al., 2014; Moses et al., 2014; Owens et al., 2011; Rosenstock et al., 2006; Russell-Jones et al., 2009; Derosa et al., 2013; Nauck et al., 2007; Schernthaner et al., 2013; Wilding et al., 2013; Kendall et al., 2005). Table S5 summarizes the raw data from the included trials. The network models were tested for consistency and the direct comparison results were similar to the indirect comparison for both HbA1c (*p* = 0.996) and body weight (*p* = 0.431).

#### Triple therapy vs. dual therapy

All classes of medicines, in combination with MET+ SU, included in the network analysis provided a significantly better and clinically relevant (>0.3%, >3.3 mmol/mol) reduction in HbA_1*c*_ when compared to MET+ SU dual therapy, with the exception of MET+ TZD+ DPP-4-i triple therapy, which did not provide a significant difference over MET+ SU (Table 3, Fig. 2A). There are no statistically significant differences with regards to change in HbA_1*c*_ for any of the comparisons with MET+ TZD or SU+ DPP-4-i (Table 3).

Only SGLT2-i and GLP-1-RA (added to MET+ SU) showed a significant reduction in body weight compared to MET+ SU dual therapy (mean difference (MD): −1.76 kg; 95% CI: −2.74 to −0.78 kg and MD: −1.55 kg; 95% CI: −2.34 to −0.74 kg respectively) (Fig. 2B); SU+ DPP-4-i dual therapy (MD: −2.89 kg; 95% CI: −4.20 to −1.59 kg and MD: −2.60 kg; 95% CI: −3.79 to −1.42 kg respectively); and MET+ TZD dual therapy (MD: −1.833 kg; 95% CI: −3.36 to −0.30 kg and MD: −1.59 kg; 95% CI: −2.95 to −0.22 kg respectively) (Table 3). Compared to MET+ SU dual therapy, triple therapy with MET+ SU+ TZD (MD: 3.5 kg; 95% CI: 2.3 to 4.6 kg) and MET+ SU+ insulin (MD: 2.5 kg; 95% CI: 1.5 to 3.4 kg) showed significant increases in body weight.

#### Triple therapy vs. triple therapy

When triple therapies are compared with each other, there are no statistically significant differences with regards to change in HbA_1*c*_ for any of the comparisons (Table 4). The only comparison which met the criteria of similar efficacy was MET+ SU+ insulin versus MET+ SU+ GLP-1-RA (MD: −0.01% (0.1 mmol/mol), 95% CI: −0.32 to 0.30% (−3.5 to 3.3 mmol/mol)).

Most triple therapies were statistically significantly different when compared to each other with respect to body weight changes (Table 4). Combined with MET+ SU, DPP-4-i, TZD, or insulin therapy produced statistically more weight gain than MET+ SU+ SGL2-i and MET+ SU+ GLP-1-RA therapy (Table 4). MET+ SU+ TZD provided significant weight gain compared to MET+ TZD+ DPP-4-i or MET+ SU+ DPP-4-i. (Table 4).

### Adverse events results

Hypoglycaemia was the only adverse event reported in the majority of the trials. Ten RCTs were identified for inclusion in the network analysis for hypoglycaemia (*N* = 4, 458) (Bergenstal et al., 2009; Dailey et al., 2004; Hermansen et al., 2007; Liu et al., 2013; Lukashevich et al., 2014; Moses et al., 2014; Owens et al., 2011; Rosenstock et al., 2006; Russell-Jones et al., 2009; Kendall et al., 2005). Data for MET+ SU+ SGLT2-i were only available for the 12 month time point (Schernthaner et al., 2013; Wilding et al., 2013) and not available at six months; therefore, this triple therapy was not included in the network analysis.

All adverse events, serious adverse events and serious hypoglycaemia were not systematically reported in the trials, and therefore no further network analyses are able to be presented.

#### Triple therapy vs. dual therapy

MET+ SU+ TZD, MET+ SU+ DPP-4-i and MET+ SU+ insulin had increased odds ratio of hypoglycaemia (Table 3, Fig. 2C), while MET+ SU+ GLP-1-RA did not significantly increase the odds ratio (1.61; 95% CI [0.90–2.87]), when compared to MET+ SU.

#### Triple therapy vs. triple therapy

There were no statistically significant differences in the odds of hypoglycaemia between most triple therapies; however, MET+ SU+ GLP-1-RA had reduced odds ratio for hypoglycaemia compared to MET+ SU+ insulin (odds ratio 0.44; 95% CI [0.25–0.76]) (Table 4).

The network model was tested for consistency and there were some differences between the direct comparison and the indirect comparison for hypoglycaemia (*p* = 0.004). The majority of inconsistency can be accounted for by Hermansen et al. (2007) (test for consistency; *p* = 0.144 with Hermansen et al. (2007) removed).

## Discussion

The results of the review and network analyses demonstrated that the addition of a third pharmaceutical class to MET+ SU therapy was statistically and clinically more effective (upper CI greater than the MCID of 0.30%, 3.3 mmol/mol) at reducing HbA_1*c*_ than dual therapy with MET+ SU. Only MET+ TZD+ DPP-4-i showed no improvement in HbA_1*c*_ when compared to MET+ SU. When comparing the different triple therapies it was difficult to identify any differences between them with regards to HbA_1*c*_, all therapies had overlapping 95% confidence intervals comparing each other. Only one comparison met the similarity criteria of confidence intervals within 0.3% (3.3 mmol/mol) of each other (MET+ SU+ GLP-1-RA vs MET+ SU+ insulin) as the statistical uncertainties were too great to draw similarity conclusions.

Most clinical guidelines advocate an individualised approach to setting patient HbA_1*c*_ targets which should be achieved for optimal treatment of type 2 diabetes (American Diabetes Association, 2014; Inzucchi et al., 2015; Canadian Agency for Drugs and Technologies in Health , 2013; Gunton et al., 2014; National Institute for Health and Clinical Excellence, 2011; New Zealand Guidelines Group, 2011). For triple therapy, guidelines commonly recommend insulin as the preferred option in combination with metformin and sulfonylurea (American Diabetes Association, 2014; Canadian Agency for Drugs and Technologies in Health , 2013; National Institute for Health and Clinical Excellence, 2011; New Zealand Guidelines Group, 2011); however, other treatments can be used if the preferred option is not suitable for the patient due to contraindications or intolerances or individual circumstances (American Diabetes Association, 2014; Canadian Agency for Drugs and Technologies in Health , 2013; Gunton et al., 2014; National Institute for Health and Clinical Excellence, 2011; New Zealand Guidelines Group, 2011), with some guidelines expanding the goal of therapy beyond the control of hyperglycemia and suggesting a more composite target to incorperate HbA_1*c*_, body weight reduction as well as cardiovascular outcomes (Inzucchi et al., 2015; Rodbard et al., 2009). When assessing composite endpoints of HbA_1*c*_ and body weight MET+ SU+ SGLT2-i and MET+ SU+ GLP-1-RA were more efficient at reducing HbA_1*c*_ levels and bodyweight than other treatments when compared to MET+ SU. While other combinations tended to be effective at reducing HbA_1*c*_ they were either no different than MET+ SU at reducing weight (MET+ SU+ TZD) or increased weight when compared to MET+ SU (MET+ SU+ DPP-4-i, MET+ SU+ insulin and MET+ SU+ TZD).

MET+ SU+ insulin had higher hypoglycaemia events than MET+ SU+ GLP-1-RA. Overall, the systematic literature review could identify only limited evidence on the long-term safety of type 2 diabetes medicines when used in triple therapy because the longest trial was 1 year. While there are longer term dual therapy studies for some of the combinations, this is of concern considering that these medicines are intended for long-term use.

It is important to note that different drugs of the same therapy class were pooled in the analysis. The assumptions were made that treatments within therapy classes had similar mechanisms of action and hence efficacy. For example; the insulin group contained long-acting basal insulin analogue (glargine) (Heine et al., 2005; Rosenstock et al., 2006; Russell-Jones et al., 2009) and fast-acting insulin analogue (aspart) (Bergenstal et al., 2009; Nauck et al., 2007), and these were grouped together in the analysis. However, this would have limited effect on the overall outcomes as comparative studies between these groups have shown that glargine and aspart are non-inferior and have similar safety margins (Strojek et al., 2009; Yang et al., 2013). There are also numerous studies that suggest that the different drugs within each therapy class included in the network meta-analysis have similar efficacy and safety outcomes; DPP-4-i (Deacon, 2011), GLP-1-RA (Rigato & Fadini, 2014), and TZD (Norris, Carson & Roberts, 2007). There was only one SGLT2-i triple therapy drug combination available, so data for other SGLT2-i triple therapy was not assessed.

There were some limitations to the analysis of these trials and these should be taken into consideration when assessing the outcomes of the network analyses. The majority of trials were powered only to assess HbA_1*c*_ and had a short duration of 24–30 weeks and the network meta-analyses were done for this time point. The number of participants was generally low and the adverse event rates were also low, for this reason it is difficult to draw conclusions on the long-term effect that these therapies will have on the incidences of adverse events. Also it was not always clear which outcomes occurred but were not reported, or whether they did not occur in the trial period.

Performing a network meta-analysis, which uses indirect comparison analyses, may introduce statistical uncertainty; therefore we applied a random effect model to allow for heterogeneity between studies to be incorporated into the estimates of effect. We also tested the network for consistency to evaluate disagreement between direct and indirect evidence, to ensure that the interpretation of the network model was consistent with the original trials.

There are limitations with the available data as there were only three trials that do not include MET + SU as the baseline dual therapy. The network model for hypoglycaemia demonstrated some inconsistency between the direct comparisons and the indirect comparison. There could be a number of reasons for this including differences in the definitions of a hypoglycaemic event and some trials allowing dose titration when an event occurred. In the network, the majority of difference could be accounted for by one trial Hermansen et al. (2007). The definitions for hypoglycaemia were different between trials, and Hermansen et al. (2007) did not report any definition. This could eventuate in the Hermansen et al. (2007) trial measuring different hypoglycaemic severity than the other trials. There were fewer trials providing data on hypoglycaemia and this could lead also to an increase in the statistical uncertainty.

## Conclusions

The network meta-analysis highlights areas where the individualised approach can be used. As examples MET+ SU+ GLP-1-RA or MET+ SU+ SGLT2-i may be a good choice for patients that require weight loss and MET+ SU+ GLP-1-RA for patients at risk for hypoglycaemia, whereas MET+ SU+ insulin may provide good blood glucose control in patients where hypoglycaemia and weight gain is not a concern. Care when choosing a triple therapy combination is needed as there is often a risk of increased hypoglycaemia events associated with this regimen, and there are very limited data surrounding the long-term effectiveness and safety of combined therapies.

## Supplemental Information

10.7717/peerj.1461/supp-1 Supplemental Information 1 Outcome data from included trials for meta analysis

10.7717/peerj.1461/supp-2 Supplemental Information 2 Supplementary figures and tables

10.7717/peerj.1461/supp-3 Supplemental Information 3 PRISMA Checklist PRISMA Checklist

10.7717/peerj.1461/supp-4 Supplemental Information 4 Systematic review rational

## Abbreviations

DPP-4-i: Dipeptidyl peptidase 4 inhibitors (also known as ‘gliptins’)
FDA: Federal Drug Agency
GLP-1-RA: Glucagon-like peptide-1 agonist (also known as incretin analogues)
MCID: Minimum clinically important difference
MD: Mean difference
MET: Metformin
OR: Odds ratio
PRISMA: Preferred Reporting Items for Systematic Reviews and Meta-Analyses
RCT: Randomised Control Trial
SGLT2-i: Sodium-glucose linked transporter protein 2 inhibitor
SU: Sulfonylurea
TZD: Thiazolidinedione

## Additional Information and Declarations

### Data Availability

The following information was supplied regarding data availability: The research in this article did not generate any raw data. The data used in this review was extracted from published articles, and an excel file with this data is included as Supplemental Information 1.
