# Supplementary material for: Triple therapy in type 2 diabetes; a systematic review and network meta-analysis
Source: PeerJ. 2015 Dec 7;3:e1461. doi: 10.7717/peerj.1461 (PMC4675096; doi:10.7717/peerj.1461)
Supplement: Supplemental Information 2 [file peerj-03-1461-s002.docx]

# Suplementary data

Contents

[SYSTEMATIC LITERATURE REVIEW 1](#_Toc421798120)

[Stage 1: Search Terms For Systematic Reviews 1](#_Toc421798121)

[Stage 2: Systematic literature review – update from identified reviews 4](#_Toc421798122)

[Statistical Analyses 7](#_Toc421798123)

[Results 10](#_Toc421798124)

[Excluded trials 10](#_Toc421798125)

[Table 1: Excluded treatment arms and studies 10](#_Toc421798126)

[Risk of bias 11](#_Toc421798127)

[Table 2: Key features and assessment of bias of the triple therapy trials. 11](#_Toc421798128)

[Eligibility criteria 13](#_Toc421798129)

[Table 3: Eligibility criteria in the triple therapy trials 13](#_Toc421798130)

[Baseline characteristics 16](#_Toc421798131)

[Table 4: Key baseline characteristics from the triple therapy trials 16](#_Toc421798132)

[Results of Efficacy and adverse events in included trials 21](#_Toc421798133)

[Table 5: Results of the triple therapy trials 21](#_Toc421798134)

# SYSTEMATIC LITERATURE REVIEW

## Stage 1: Search Terms For Systematic Reviews

*Ovid Medline (R)*

Ovid MEDLINE(R) In-Process & Other Non-Indexed Citations, Ovid MEDLINE(R) Daily and Ovid MEDLINE(R) 1946 to 5^th^ March 2014

1. metformin.mp.

2. glucophage.mp.

3. dimethylbiguanidine.mp.

4. dimethylguanylguanidine.mp.

5. exp Metformin/

6. (Fortamet or Glucophage or Glucophage XR or Glumetza or Riomet).mp.

7. 1 or 2 or 3 or 4 or 5 or 6

8. exp Sulfonylurea Compounds/

9. Gliclazide.mp.

10. Glimepiride.mp.

11. Glipizide.mp

12. Glibenclamide.mp

13. glyburide.mp.

14. (Glucotrol or Diamicron or Glyade or Nidem or Glimel or Daonil or Aylide or Diapride or Dimirel or Amaryl).mp.

15. Sulfonylurea*.mp.

16. 8 or 9 or 10 or 11 or 12 or 13 or 14 or 15

17. exp Thiazolidinediones/

18. pioglitazone*.mp.

19. rosiglitazone*.mp.

20. Thiazolidinedione*.mp.

21. (Acpio or Actos or Pizaccord or Prioten or Vexazone or Avandia or Avandamet).mp.

22. 17 or 18 or 19 or 20 or 21

23. exp alpha-Glucosidases/ai

24. Acarbose.mp or exp Acarbose/

25. Glucobay.mp.

26. 23 or 24 or 25

27. exp Dipeptidyl-Peptidase IV Inhibitors/

28. Alogliptin.mp.

29. Sitagliptin.mp.

30. Saxagliptin.mp.

31. Linagliptin.mp

32. Vildagliptin.mp.

33. (Nesina or Juvicor or Januvia or Janumet or Onglyza or Kombiglyze or Trajenta or Trajentamet or Galvus or Galvumet).mp.

34. (Dipeptidyl-Peptidase IV Inhibitor* or Dipeptidyl-Peptidase 4 Inhibitor*or DPP-4 inhibitor* or DPP4 inhibitor* or DPP-IV inhibitor* or DPPIV inhibitor*).mp.

35. 27 or 28 or 29 or 30 or 31 or 32 or 33 or 34

36. exp Glucagon-Like Peptide 1/

37. Incretin Mimetic*.mp.

38. Glucagon-Like Peptide 1.mp.

39. GLP-1.mp.

40. Exenatide.mp.

41. Liraglutide.mp.

42. (Byetta or Victoza or Bydureon).mp.

43. 36 or 37 or 38 or 39 or 40 or 41 or 42

44. exp Insulin, Short-Acting/ or exp Insulin, Regular, Pork/ or exp Insulin, Long-Acting/ or exp Insulin, Regular, Human/

45. Aspart.mp.

46. Lispro.mp.

47. Glulisine.mp

48. Insulin Neutral.mp

49. Detemir.mp.

50. Glargine.mp.

51. (Isophane or NPH or neutral protamine Hagedorn).mp.

52. (Novorapid or NovoMix or Humalog or Apidra or Actrapid or Humulin or Levemir or Lantus or Protaphane or Mixtard or Hypurin Neutral).mp

53. 44 or 45 or 46 or 47 or 48 or 49 or 50 or 51 or 52

54. exp Sodium-Glucose Transporter 2/ai

55. SGLT2 Inhibitor*.mp

56. Sodium-glucose co-transporter-2 inhibitor*.mp.

57. Sodium-glucose transporter-2 inhibitor*.mp

58. Canagliflozin.mp.

59. Dapagliflozin.mp.

60. (Invokana or Forxiga or Farxiga).mp.

61. 54 or 55 or 56 or 57 or 58 or 59 or 60

62. 7 or 16 or 22 or 26 or 35 or 43 or 53 or 61

63. exp Diabetes Mellitus, Type 2/

64. Diabetes Mellitus Type 2.mp.

65. Diabetes Mellitus Type II.mp.

66. DM type 2.mp.

67. DM type II.mp.

68. Type 2 Diabetes.mp.

69. Type II Diabetes.mp.

70. Diabetes Type II.mp.

71. Diabetes Type 2.mp

72. T2DM.mp.

73. DMT2.mp.

74. 63 or 64 or 65 or 66 or 67 or 68 or 69 or 70 or 71 or 72 or 73

75. systematic review*.mp.

76. meta analysis.mp

77. exp Meta-Analysis/

78. 75 or 76 or 77

79. 62 and 74 and 78

80. limit 79 to (english language and yr="2010 -Current")

*The Cochrane Library*

Search conducted on the 5^th^ March 2014

ID Search

#1 metformin

#2 glucophage

#3 dimethylbiguanidine

#4 dimethylguanylguanidine

#5 MeSH descriptor: [Metformin] explode all trees

#6 Fortamet or Glucophage or Glucophage XR or Glumetza or Riomet

#7 [#27-#6]

#8 MeSH descriptor: [Sulfonylurea Compounds] explode all trees

#9 Gliclazide

#10 Glimepiride

#11 Glipizide

#12 Glibenclamide

#13 glyburide

#14 Glucotrol or Diamicron or Glyade or Nidem or Glimel or Daonil or Aylide or Diapride or Dimirel or Amaryl

#15 Sulfonylurea*

#16 [#39-#15]

#17 MeSH descriptor: [Thiazolidinediones] explode all trees

#18 pioglitazone*

#19 rosiglitazone*

#20 Thiazolidinedione*

#21 Acpio or Actos or Pizaccord or Prioten or Vexazone or Avandia or Avandamet

#22 [#30-#21]

#23 alpha-glucosidase inhibitor

#24 Acarbose

#25 MeSH descriptor: [Acarbose] explode all trees

#26 Glucobay

#27 (or #23-#26)

#28 MeSH descriptor: [Dipeptidyl-Peptidase IV Inhibitors] explode all trees

#29 Alogliptin

#30 Sitagliptin

#31 Saxagliptin

#32 Linagliptin

#33 Vildagliptin

#34 Nesina or Juvicor or Januvia or Janumet or Onglyza or Kombiglyze or Trajenta or Trajentamet or Galvus or Galvumet

#35 Dipeptidyl-Peptidase IV Inhibitor* or Dipeptidyl-Peptidase 4 Inhibitor*or DPP-4 inhibitor* or DPP4 inhibitor* or DPP-IV inhibitor* or DPPIV inhibitor*

#36 [#40-#35]

#37 MeSH descriptor: [Glucagon-Like Peptide 1] explode all trees

#38 Incretin Mimetic

#39 Glucagon-Like Peptide 1

#40 GLP-1

#41 Exenatide

#42 Liraglutide

#43 Byetta or Victoza or Bydureon

#44 (or #37-#43)

#45 MeSH descriptor: [Insulin, Short-Acting] explode all trees

#46 MeSH descriptor: [Insulin, Regular, Human] explode all trees

#47 MeSH descriptor: [Insulin, Long-Acting] explode all trees

#48 MeSH descriptor: [Insulin, Regular, Pork] explode all trees

#49 Aspart

#50 Lispro

#51 Glulisine

#52 Insulin Neutral

#53 Detemir

#54 Glargine

#55 Isophane or NPH or neutral protamine Hagedorn

#56 Novorapid or NovoMix or Humalog or Apidra or Actrapid or Humulin or Levemir or Lantus or Protaphane or Mixtard or Hypurin Neutral

#57 [#41-#56]

#58 MeSH descriptor: [Sodium-Glucose Transporter 2] explode all trees

#59 SGLT2 Inhibitor*

#60 Sodium-glucose co-transporter-2 inhibitor*

#61 Sodium-glucose transporter-2 inhibitor*

#62 Canagliflozin

#63 Dapagliflozin

#64 Invokana or Forxiga or Farxiga

#65 [#32-#64]

#66 #7 or #16 or #22 or #27 or #36 or #44 or #57 or #65

#67 MeSH descriptor: [Diabetes Mellitus, Type 2] explode all trees

#68 Diabetes Mellitus Type 2

#69 Diabetes Mellitus Type II

#70 DM type 2

#71 DM type II

#72 Type 2 Diabetes

#73 Type II Diabetes

#74 Diabetes Type II

#75 Diabetes Type 2

#76 T2DM

#77 DMT2

#78 [#33-#77]

#79 #66 and #78 from 2010

## Stage 2: Systematic literature review – update from identified reviews

*Bennett et al., 2011 Update*

*Ovid Medline (R)*

Ovid MEDLINE(R) 1946 to Present with Daily Update, Database Field Guide & Ovid MEDLINE(R) In-Process & Other Non-Indexed Citations October 06, 2014

ID Search

1. exp thiazolidinediones/ or exp glipizide/ or exp glyburide/ or exp metformin/ or exp acarbose/ or (thiazolidinedione or pioglitazone or rosiglitazone or sulfonylurea* or sulphonylurea* or glipizide or glyburide or glimepiride or glibenclamide or biguanide* or metformin or insulin secretagogues or meglitinide* or repaglinide or nateglinide or alpha-glucosidase inhibitors or alpha-glucosidase inhibitor or acarbose or Dipeptidyl-Peptidase IV Inhibitors or sitagliptin* or saxagliptin* or dpp-4 or dpp-iv or liraglutide or exenatide or bromocriptine or colesevelam).ti,ab. or exp bromocriptine/ or exp Glucagon-Like Peptide 1/

2. limit 1 to yr="2010 -Current"

3. exp insulin/ or (long acting insulin* or long acting analog* or slow* acting insulin* or slow* acting analog* or nph insulin or humulin or novolin or glargine or Lantus or Optisulin or hoe 901 or 160337-95-1 or detemir or determir or Levemir or nn 304 or 169148-63-4 or 11061-68-0 or Lispro or Lyspro or Humalog or Liprolog or 133107-64-9 or Insulin Aspart or 116094-23-6 or NovoLog or NovoRapid or NovoMix or Glulisine or 207748-29-6 or Apidra).ti,ab,rn. or (short acting insulin* or quick acting insulin* or rapid acting insulin* or rapidly acting insulin* or fast acting insulin* or quick acting analog* or rapid acting analog* or rapidly acting analog* or short acting analog* or fast acting analog*).ti,ab.

4. limit 3 to yr="2002 -Current"

5. (exp Diabetes Mellitus, Type 2/ or (diabet* and (non-insulin dependent or type-2 or type II or type 2)).ti,ab.) and English.lg.

6. (Randomized Controlled Trial.pt. or exp Randomized Controlled Trials as Topic/ or exp Randomized Controlled Trial/ or RCT*.mp.) not (exp animal/ not exp human/) [mp=title, abstract, original title, name of substance word, subject heading word, keyword heading word, protocol supplementary concept word, rare disease supplementary concept word, unique identifier]

7. 2 or 4

8. 5 and 6 and 7

*The Cochrane Library*

Search conducted on the 8^th^ Oct 2014

ID Search

#1 MeSH descriptor: [Metformin] explode all trees

#2 MeSH descriptor: [Sulfonylurea Compounds] explode all trees

#3 thiazolidinedione* or pioglitazone or rosiglitazone or sulfonylurea* or sulphonylurea* or glipizide or glyburide or glimepiride or glibenclamide or biguanide* or metformin or "insulin secretagogues" or meglitinide* or repaglinide or nateglinide or "alpha-glucosidase inhibitors" or "alpha-glucosidase inhibitor" or acarbose or "Dipeptidyl-Peptidase IV Inhibitors" or saxagliptin* or sitagliptin* or liraglutide or exenatide or bromocriptine or colesevelam

#4 (diabetes near type-2) or (diabet*:ti,ab,kw and ("non-insulin dependent":ti,ab,kw or type-2:ti,ab,kw or "type II":ti,ab,kw or "type 2":ti,ab,kw))

#5 MeSH descriptor: [Diabetes Mellitus, Type 2] explode all trees

#6 (#1 or #2 or #3) Publication Year from 2010

#7 #4 or #5

#8 MeSH descriptor: [Insulin, Short-Acting] explode all trees

#9 MeSH descriptor: [Insulin, Regular, Human] explode all trees

#10 MeSH descriptor: [Insulin, Long-Acting] explode all trees

#11 MeSH descriptor: [Insulin, Regular, Pork] explode all trees

#12 Aspart or Lispro or Glulisine or Insulin Neutral or Detemir or Glargine or Isophane or NPH or neutral protamine Hagedorn

#13 Novorapid or NovoMix or Humalog or Apidra or Actrapid or Humulin or Levemir or Lantus or Protaphane or Mixtard or Hypurin Neutral

#14 [#9-#13] Publication Year from 2002

#15 (#6 or #14) and #7 in Trials

*Berhan 2013 (SGLT-2 inhibitors) Update*

*Ovid Medline (R)*

Ovid MEDLINE(R) 1946 to Present with Daily Update, Database Field Guide & Ovid MEDLINE(R) In-Process & Other Non-Indexed Citations October 06, 2014

ID Search

1 ((exp Sodium-Glucose Transporter 2/ai or (SGLT2 Inhibitor* or Sodium-glucose co-transporter-2 inhibitor* or Sodium-glucose transporter-2 inhibitor* or Canagliflozin or Dapagliflozin or Invokana or Forxiga or Farxiga).mp.) and (exp Diabetes Mellitus, Type 2/ or (diabet* and (non-insulin dependent or type-2 or type II or type 2)).ti,ab.) and English.lg. and (Randomized Controlled Trial.pt. or exp Randomized Controlled Trials as Topic/ or exp Randomized Controlled Trial/ or RCT*.mp.)) not (exp animal/ not exp human/)

2 limit 1 to yr="2013 -Current"

*The Cochrane Library*

Search conducted on the 8^th^ Oct 2014

ID Search

#1 SGLT2 Inhibitor* or Sodium-glucose co-transporter-2 inhibitor* or Sodium-glucose transporter-2 inhibitor* or Canagliflozin or Dapagliflozin or Invokana or Forxiga or Farxiga

#2 (diabetes near type-2) or (diabet*:ti,ab,kw and ("non-insulin dependent":ti,ab,kw or type-2:ti,ab,kw or "type II":ti,ab,kw or "type 2":ti,ab,kw))

#3 MeSH descriptor: [Diabetes Mellitus, Type 2] explode all trees

#4 (#1) and (#2 or #3) from 2013, in Trials

*Monami 2010 (DPP-4-i) Update*

*Ovid Medline (R)*

Ovid MEDLINE(R) 1946 to Present with Daily Update, Database Field Guide & Ovid MEDLINE(R) In-Process & Other Non-Indexed Citations October 06, 2014

ID Search

1. ((exp Dipeptidyl-Peptidase IV Inhibitors/ or (Alogliptin or Sitagliptin or Saxagliptin or Linagliptin or Vildagliptin or Nesina or Juvicor or Januvia or Janumet or Onglyza or Kombiglyze or Trajenta or Trajentamet or Galvus or Galvumet or Dipeptidyl-Peptidase IV Inhibitor* or Dipeptidyl-Peptidase 4 Inhibitor*or DPP-4 inhibitor* or DPP4 inhibitor* or DPP-IV inhibitor* or DPPIV inhibitor*).mp.) and (exp Diabetes Mellitus, Type 2/ or (diabet* and (non-insulin dependent or type-2 or type II or type 2)).ti,ab.) and English.lg. and (Randomized Controlled Trial.pt. or exp Randomized Controlled Trials as Topic/ or exp Randomized Controlled Trial/ or RCT*.mp.)) not (exp animal/ not exp human/)

2. limit 1 to yr="2008 -Current"

*The Cochrane Library*

Search conducted on the 8^th^ Oct 2014

ID Search

#1 MeSH descriptor: [Dipeptidyl-Peptidase IV Inhibitors] explode all trees

#2 Alogliptin or Sitagliptin or Saxagliptin or Linagliptin or Vildagliptin or Nesina or Juvicor or Januvia or Janumet or Onglyza or Kombiglyze or Trajenta or Trajentamet or Galvus or Galvumet or Dipeptidyl-Peptidase IV Inhibitor* or Dipeptidyl-Peptidase 4 Inhibitor*or DPP-4 inhibitor* or DPP4 inhibitor* or DPP-IV inhibitor* or DPPIV inhibitor*

#3 (diabetes near type-2) or (diabet*:ti,ab,kw and ("non-insulin dependent":ti,ab,kw or type-2:ti,ab,kw or "type II":ti,ab,kw or "type 2":ti,ab,kw))

#4 MeSH descriptor: [Diabetes Mellitus, Type 2] explode all trees

#5 (#1 or #2) and (#3 or #4) from 2008, in Trials

# Statistical Analyses

## HbA_1c_ STATA do file:

display _newline(5)

display "syntax executed at " c(current_time) " on " c(current_date)

* * * * * * * * * * * * * * * *|* * * * * * * * * * * * * * * *

* *

* DIABETES T2 MEDICATION *

* Syntax description *

* *

* * * * * * * * * * * * * * * *|* * * * * * * * * * * * * * * *

*set output error // remove * when output starts to get too long

clear all

set more off // chose which one more on

version 12

set linesize 80

set varabbrev off, permanently

set autotabgraphs on, permanently

/* SYNTAX REVISION HISTORY (include date and name)

created - 22/08/2014

modified - 20/06/2015

*/

cd ""

set output proc

* ----------------------------------------------------------------------------

* O O O O O O O O O O O O O O O O O O O

* ----------------------------------------------------------------------------

. import excel "Raw for repository.xlsx", sheet("HbA1c") firstrow

//Label variables

. label variable nA "MET+SU+PBO"

. label variable bA "MET+SU+PBO"

. label variable vA "MET+SU+PBO"

. label variable nB "MET+SU+GLP-1-RA"

. label variable bB "MET+SU+GLP-1-RA"

. label variable vB "MET+SU+GLP-1-RA"

. label variable nC "MET+SU+INS"

. label variable bC "MET+SU+INS"

. label variable vC "MET+SU+INS"

. label variable nD "MET+SU+TZD"

. label variable bD "MET+SU+TZD"

. label variable vD "MET+SU+TZD"

. label variable nE "MET+SU+DPP-4-i"

. label variable bE "MET+SU+DPP-4-i"

. label variable vE "MET+SU+DPP-4-i"

. label variable nF "SU+DPP-4-i"

. label variable bF "SU+DPP-4-i"

. label variable vF "SU+DPP-4-i"

. label variable nG "MET+TZD+DPP-4-i"

. label variable bG "MET+TZD+DPP-4-i"

. label variable vG "MET+TZD+DPP-4-i"

. label variable nH "MET+TZD+PBO"

. label variable bH "MET+TZD+PBO"

. label variable vH "MET+TZD+PBO"

. label variable nI "MET+SU+SGLT2-i"

. label variable bI "MET+SU+SGLT2-i"

. label variable vI "MET+SU+SGLT2-i"

//setup network

network setup b v n, studyvar(study) md

// Run consistency model network

network meta c

// test for best intervention

mvmeta, pbest(max in 1, zero reps(9000)) noest

// run inconsistency model

network meta i

//test for inconsistency

network forest, l

// remove files before activating save -

graph save Graph "Consistency Plots.gph"

//Convert to pairs for plot

network convert pairs

//Plot

networkplot _t1 _t2, lab (MET+SU MET+SU+GLP-1-RA MET+SU+INS MET+SU+TZD MET+SU+DPP-4-i SU+DPP-4-i MET+TZD+DPP-4-i MET+TZD MET+SU+SGLT2-i) tit (Triple Therapy HbA1c)

// remove files before activating save -

graph save Graph "Network Diagram.gph"

* HEADER

// sub-header

* ----------------------------------------------------------------------------

* Re-order Drugs

* ----------------------------------------------------------------------------

import excel "Raw for repository.xlsx", sheet("Sheet1") firstrow clear

// reorder first to last

order nA bA vA, last

//setup network

network setup b v n, studyvar(study) md

// Run consistency model network

network meta c

* ----------------------------------------------------------------------------

* Re-order Drugs

* ----------------------------------------------------------------------------

import excel "Raw for repository.xlsx", sheet("Sheet1") firstrow clear

// reorder first to last

order nA bA vA, last

// reorder first to last

order nB bB vB, last

//setup network

network setup b v n, studyvar(study) md

// Run consistency model network

network meta c

* ----------------------------------------------------------------------------

* Re-order Drugs

* ----------------------------------------------------------------------------

import excel "Raw for repository.xlsx", sheet("Sheet1") firstrow clear

// reorder first to last

order nA bA vA, last

// reorder first to last

order nB bB vB, last

// reorder first to last

order nC bC vC, last

//setup network

network setup b v n, studyvar(study) md

// Run consistency model network

network meta c

* ----------------------------------------------------------------------------

* Re-order Drugs

* ----------------------------------------------------------------------------

import excel "Raw for repository.xlsx", sheet("Sheet1") firstrow clear

// reorder first to last

order nA bA vA, last

// reorder first to last

order nB bB vB, last

// reorder first to last

order nC bC vC, last

// reorder first to last

order nD bD vD, last

//setup network

network setup b v n, studyvar(study) md

// Run consistency model network

network meta c

* ----------------------------------------------------------------------------

* Re-order Drugs

* ----------------------------------------------------------------------------

import excel "Raw for repository.xlsx", sheet("Sheet1") firstrow clear

// reorder first to last

order nA bA vA, last

// reorder first to last

order nB bB vB, last

// reorder first to last

order nC bC vC, last

// reorder first to last

order nD bD vD, last

// reorder first to last

order nE bE vE, last

//setup network

network setup b v n, studyvar(study) md

// Run consistency model network

network meta c

* ----------------------------------------------------------------------------

* Re-order Drugs

* ----------------------------------------------------------------------------

import excel "Raw for repository.xlsx", sheet("Sheet1") firstrow clear

// reorder first to last

order nA bA vA, last

// reorder first to last

order nB bB vB, last

// reorder first to last

order nC bC vC, last

// reorder first to last

order nD bD vD, last

// reorder first to last

order nE bE vE, last

// reorder first to last

order nF bF vF, last

//setup network

network setup b v n, studyvar(study) md

// Run consistency model network

network meta c

* ----------------------------------------------------------------------------

* Re-order Drugs

* ----------------------------------------------------------------------------

import excel "Raw for repository.xlsx", sheet("Sheet1") firstrow clear

// reorder first to last

order nA bA vA, last

// reorder first to last

order nB bB vB, last

// reorder first to last

order nC bC vC, last

// reorder first to last

order nD bD vD, last

// reorder first to last

order nE bE vE, last

// reorder first to last

order nF bF vF, last

// reorder first to last

order nG bG vG, last

//setup network

network setup b v n, studyvar(study) md

// Run consistency model network

network meta c

* ----------------------------------------------------------------------------

* Re-order Drugs

import excel "Raw for repository.xlsx", sheet("Sheet1") firstrow clear

// reorder first to last

order nA bA vA, last

// reorder first to last

order nB bB vB, last

// reorder first to last

order nC bC vC, last

// reorder first to last

order nD bD vD, last

// reorder first to last

order nE bE vE, last

// reorder first to last

order nF bF vF, last

// reorder first to last

order nG bG vG, last

// reorder first to last

order nH bH vH, last

//setup network

network setup b v n, studyvar(study) md

// Run consistency model network

network meta c

* ----------------------------------------------------------------------------

* end of Syntax01

# Results

### Figure 1. Flow diagram showing the total number of records identified and the number of records filtered at each stage of the selection process from the systematic search for systematic reviews of type 2 diabetes in March 2014.


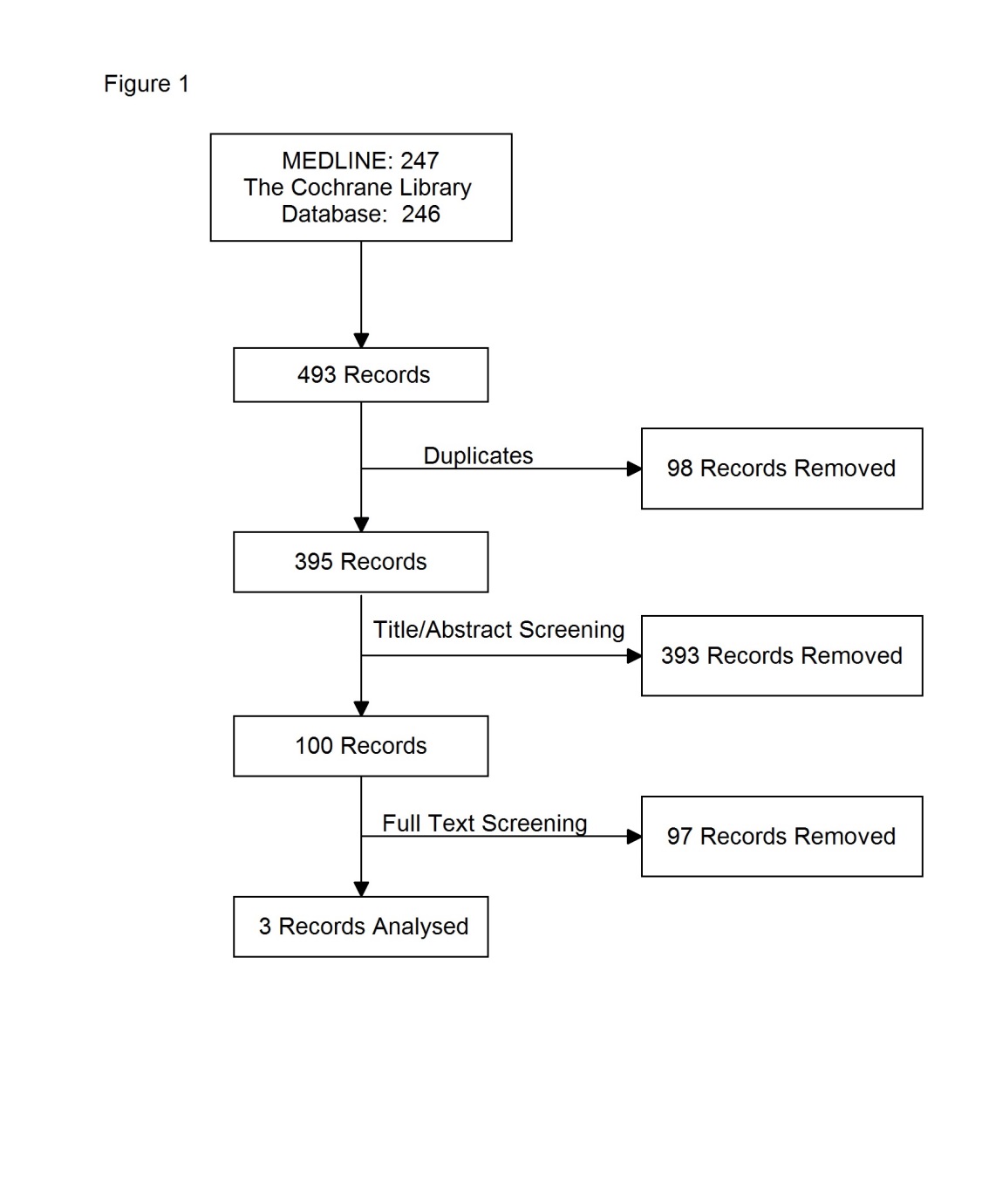


Figure 2. Evidence network of direct comparisons for outcomes HbA1c and body weight in a systematic review and network meta-analysis for triple therapy in type 2 diabetes.
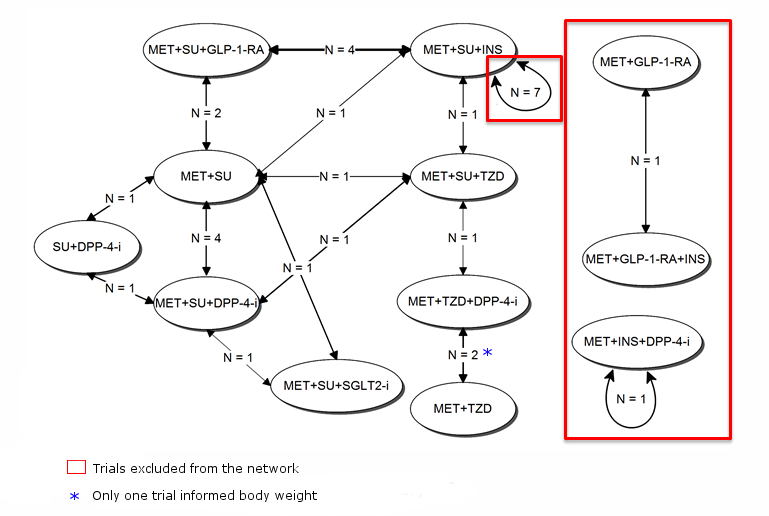


## Excluded trials

### Table 1: Excluded treatment arms and studies

| Intervention 1 | Intervention 2 | Number of trials | Trials | Duration  (months) | N | HbA_1c_ | BW | AE | SAE | HypoG |
| --- | --- | --- | --- | --- | --- | --- | --- | --- | --- | --- |
| MET+SU+INS | MET+SU+INS | 7 | Al-Shaikh 2006 [1] | 26 | 221 | x | x |  |  |  |
|  |  |  | Bergenstal 2009^#^ [2] | 24 | 372 | x | x | x | x | x |
|  |  |  | Esposito 2008 [3] | 36 | 116 | x | x | x | x | x |
|  |  |  | Holman 2007 [4] | 52 | 708 | x | x | x |  | x |
|  |  |  | Janka 2005 [5] | 24 | 371 | x | x | x |  | x |
|  |  |  | Strojek 2009 [6] | 26 | 469 | x | x | x |  | x |
|  |  |  | Yang 2013 [7] | 24 | 521 | x | x | x | x | x |
| MET+INS+DPP-4-i | MET+INS+DPP-4-i | 1 | Zinman 2012 [8]  (Rodbard, 2013 [9]) | 52  104 | 1,030 | x | x | x | x | x |
| MET+GLP-1-RA+INS | MET+GLP-1-RA | 1 | DeVries 2012 [10] | 26 | 323 | x | x | x | x |  |

AE = adverse event; BW = body weight; DPP-4-i = dipeptidyl peptidase-4 inhibitor; GLP-1-RA = glucagon-like peptide-1 receptor agonist; HbA_1c_ = glycated haemoglobin; HypoG = hypoglycaemic event INS = insulin; MET = metformin; SAE = serious adverse event; SU = sulfonylurea;

^#^ One trial included three treatment arms and provided information for the comparisons MET+SU+INS and MET+SU+GLP-1-RA.

1. Al-Shaikh AR: Comparison of basal insulin added to oral agents versus twice - daily premixed insulin as initial insulin therapy for type 2 diabetes. PaK J Med Sci 2006;22:14-17

2. Bergenstal R, Lewin A, Bailey T, Chang D, Gylvin T, Roberts V, NovoLog Mix-vs.-Exenatide Study G: Efficacy and safety of biphasic insulin aspart 70/30 versus exenatide in subjects with type 2 diabetes failing to achieve glycemic control with metformin and a sulfonylurea. Curr Med Res Opin 2009;25:65-75

3. Esposito K, Ciotola M, Maiorino MI, Gualdiero R, Schisano B, Ceriello A, Beneduce F, Feola G, Giugliano D: Addition of neutral protamine lispro insulin or insulin glargine to oral type 2 diabetes regimens for patients with suboptimal glycemic control: a randomized trial. Ann Intern Med 2008;149:531-539

4. Holman RR, Thorne KI, Farmer AJ, Davies MJ, Keenan JF, Paul S, Levy JC: Addition of biphasic, prandial, or basal insulin to oral therapy in type 2 diabetes. N Engl J Med 2007;357:1716-1730

5. Janka HU, Plewe G, Riddle MC, Kliebe-Frisch C, Schweitzer MA, Yki-Jarvinen H: Comparison of basal insulin added to oral agents versus twice-daily premixed insulin as initial insulin therapy for type 2 diabetes. Diabetes Care 2005;28:254-259

6. Strojek K, Bebakar WM, Khutsoane DT, Pesic M, Smahelova A, Thomsen HF, Kalra S: Once-daily initiation with biphasic insulin aspart 30 versus insulin glargine in patients with type 2 diabetes inadequately controlled with oral drugs: an open-label, multinational RCT. Curr Med Res Opin 2009;25:2887-2894

7. Yang W, Xu X, Liu X, Yang G, Seino Y, Andersen H, Jinnouchi H: Treat-to-target comparison between once daily biphasic insulin aspart 30 and insulin glargine in Chinese and Japanese insulin-naive subjects with type 2 diabetes. Curr Med Res Opin 2013;29:1599-1608

8. Zinman B, Philis-Tsimikas A, Cariou B, Handelsman Y, Rodbard HW, Johansen T, Endahl L, Mathieu C, Investigators NNT: Insulin degludec versus insulin glargine in insulin-naive patients with type 2 diabetes: a 1-year, randomized, treat-to-target trial (BEGIN Once Long). Diabetes Care 2012;35:2464-2471

9. Rodbard HW, Cariou B, Zinman B, Handelsman Y, Philis-Tsimikas A, Skjoth TV, Rana A, Mathieu C: Comparison of insulin degludec with insulin glargine in insulin-naive subjects with Type 2 diabetes: A 2-year randomized, treat-to-target trial. Diabetic Med 2013;30:1298-1304

10. DeVries JH, Bain SC, Rodbard HW, Seufert J, D'Alessio D, Thomsen AB, Zychma M, Rosenstock J, Liraglutide-Detemir Study G: Sequential intensification of metformin treatment in type 2 diabetes with liraglutide followed by randomized addition of basal insulin prompted by A1C targets. Diabetes Care 2012;35:1446-1454

## Risk of bias

### **Table 2: Key features and assessment of bias of the triple therapy trials.**

| **Trial** | **N** | **Recruitment** | **Centres**  **Design** | **Random sequence generation** | **Allocation concealment** | **Blinding of participants and personnel** | **Blinding of outcome assessment** | **Incomplete outcome data** | **Selective reporting** | **Bias due to other issues** |
| --- | --- | --- | --- | --- | --- | --- | --- | --- | --- | --- |
| **MET + SU vs. MET + SU + DPP-4-i** | | | | | | | | | | |
| Hermansen 2007 | 441 | 2009-2010 | N. America, S. America, Europe  R, DB, PC | Low | Low | Low | Low | Low | Low | Unclear |
| Owens 2011 | 1,055 | 2008-2009 | N. America, S. America, E. Europe, Asia, Middle East  R, DB, PC | Unclear | Unclear | Low | Low | High | Low | Unclear |
| Lukashevich 2014 | 318 | NR | R, DB, PC | Low | Unclear | Low | Low | High | Low | Unclear |
| Moses 2014 | 257 | 2010-2011 | Australia, Canada, India,  Korea, Thailand, UK  R, DB, PC | Low | Low | Low | Low | Low | Low | Unclear |
| **MET + SU vs. MET + SU + TZD** | | | | | | | | | | |
| Dailey 2004 | 365 | NR | N. America  R, DB, PC | Unclear | Unclear | Low | Low | High | Unclear | Unclear |
| **MET + SU vs. MET + SU + GLP-1-RA** | | | | | | | | | | |
| Kendall 2005 | 733 | 2002-2003 | N. America  R, DB, PC | Unclear | Unclear | High | Low | High | Unclear | Unclear |
| **MET + SU vs. MET + SU + GLP-1-RA vs. MET + SU + INS** | | | | | | | | |  |  |
| Russell-Jones 2009 | 581 | 2006-2007 | N. America, S. America, W. Europe, Asia  R, DB, PC | Low | Low | Low | Low | Low | Low | Unclear |
| **MET + SU vs. MET + SU + SGLT2-i** | | | | | | | | |  |  |
| Wilding 2013 | 469 | 2010-2011 | USA, Europe  R, DB, PC | Low | Low | Low | Low | Unclear | Low | Unclear |
| **MET + SU + TZD vs. MET + SU + INS** | | | | | | | | | | |
| Rosenstock 2006 | 216 | NR | N. America  R, OL | Unclear | Unclear | High | High | Low | Unclear | Unclear |
| **MET + SU + GLP-1-RA vs. MET + SU + INS** | | | | | | | | | | |
| Bergenstal 2009 | 372 | 2005-2006 | N. America  R, OL | Low | Low | High | High | High | Low | Unclear |
| Heine 2005 | 549 | 2003-2004 | Australia, N. America, S. America, W. Europe  R, OL | Low | Low | High | High | High | Low | Unclear |
| Nauck 2007 | 501 | 2003-2005 | W. Europe, E. Europe, Asia  R, OL | Low | Low | High | High | High | Low | Unclear |
| **MET + SU + SGLT2-i vs. MET + SU + DPP-4-i** | | | | | | | | | | |
| Schernthaner 2013 | | 2010-2012 | Worldwide  R, DB | Low | Low | Low | Low | High | Low | Unclear |
| **MET + SU + TZD vs. MET + SU + DPP-4-i** | | | | | | | | | | |
| Liu 2013 | 120 | 2009-2011 | Taiwan  R, OL | Low | Low | High | High | Low | Low | Low |
| **MET + TZD vs. MET + TZD + DPP-4-i** | | | | | | | | | | |
| Bosi 2011 | 803 | 2007-2009 | N. America  R, DB | Unclear | Unclear | Low | Low | Low | Low | Unclear |
| DeFronzo 2012 | 1,554 | 2006-2008 | Australia, NZ, America, Europe, Asia, Africa, M. East  R, DB, PC | Unclear | Unclear | Low | Low | High | Low | Unclear |
| **MET + TZD + SU vs. MET + TZD + DPP-4-i** | | | | | | | | | | |
| DeRosa 2013 | 453 | NR | W. Europe  R, DB | Low | Low | Low | Low | Low | Unclear | Low |

**Random sequence generation** - biased allocation to interventions due to inadequate randomisation. **Allocation concealment** - biased allocation to interventions due to inadequate concealment of allocations prior to assignment. **Blinding of participants and personnel** - performance bias due to knowledge of the allocated interventions by participants and personnel during the study. **Blinding of outcome assessment** - detection bias due to knowledge of the allocated interventions by outcome assessors. **Incomplete outcome data** - attrition bias due to amount, nature or handling or incomplete outcome data. **Selective reporting** - reporting bias due to selective outcome reporting. **Bias due to problems not covered above** – e.g. study has been claimed to be fraudulent.

## Eligibility criteria

### Table 3**: Eligibility criteria in the triple therapy trials**

| **Trial** | **Inclusion criteria** | **Exclusion criteria** |
| --- | --- | --- |
| **MET + SU vs. MET + SU + DPP-4-i** | | |
| Hermansen 2007 | - 18 - 75 years.  - Patients had to be either:  (i) Taking glimepiride alone (at any dose) or in combination with MET (at any dose); or  (ii) Taking another OAD in mono-, dual- or triple-therapy; or  (iii) Not taking any OADs in the 8 weeks prior to screening. | - T1DM.  - Treated with INS within 8 weeks of screening.  - Renal dysfunction (CrCl < 45 mL/min or < 60 mL/min if on MET). |
| Owens 2011 | - 18 - 80 years.  - HbA_1c_ of 7.0 - 10.0%.  - BMI ≤ 40 kg/m^2^.  - Treated with MET (≥ 1,500 mg/day, or the maximum tolerated daily dose) and an SU (maximum tolerated dose) at stable doses for > 10 weeks prior to screening. | - A clinical condition that would interfere with participation and safety.  - MI, stroke or transient ischaemic attack within 6 months of screening.  - Impaired hepatic function, renal failure or impairment, acute or chronic metabolic acidosis, hereditary galactose intolerance.  - Pregnancy or breastfeeding.  - Treatment with other antidiabetic agents within 3 months of screening. |
| Lukashevich 2014 | - 18 - 80 years.  - HbA_1c_ of 7.5 - 11.0%.  - BMI ≥22 to ≤ 45 kg/m^2^.  Treated with MET ± SU | - FPG ≥15.0 mmol/l.  - Hepatic, renal or cardiovascular medical conditions.  - Significant laboratory abnormalities.  - Pregnant or lactating females. |
| Moses 2014 | ≥18 years old.  BMI ≤40 kg/m^2^.  HbA1c 7.0–10.0%.  On combination therapy. | Creatinine clearance (CrCl) <1.0ml/s or creatinine kinase ≥10 times upper limit of normal.  Congestive heart failure.  Active liver disease and/or significant abnormal liver function.  History of haemoglobinopathies.  History of alcohol abuse or drug abuse ≤12 months. Use of oral antidiabetic agents other than metformin and sulphonylureas.  Treatment with systemic glucocorticoids.  Pregnant or breast-feeding. |
| **MET + SU vs. MET + SU + TZD** | | |
| Dailey 2004 | 20 - 78 years.  HbA_1c_ of 7.0 - 10.0%.  BMI of 23-40 kg/m^2^.  Treated with a stable dose of OAD for 8 weeks prior to screening. | - Uncontrolled diabetes (HbA_1c_ > 10%).  - Polyuria and polydipsia with > 10% weight loss.  - Renal dysfunction, abnormal liver function, anaemia, clinically substantial CVD or psychiatric disease, or long-term insulin therapy. |
| **MET + SU vs. MET + SU + GLP-1-RA** | | |
| Kendall 2005 | 22 - 77 years.  HbA_1c_ of 7.5 - 11.0%.  BMI of 27 - 45 kg/m^2^; Stable weight.  Treatment with MET (≥ 1,500 mg/day) and a SU (maximally effective dose) for ≥ 3 months prior to screening.  FPG < 13.3 mmol/L.  No clinically relevant abnormal laboratory test values.  Females were postmenopausal, surgically sterile or using contraceptives. | Other clinically significant medical conditions.  Use of TZDs, meglitinides, α-glucosidase inhibitors, exogenous INS or weight loss drugs in the prior 3 months.  Therapy with corticosteroids, drugs that effect GIT motility, transplantation medication or any investigational drug. |
| **MET + SU vs. MET + SU + GLP-1-RA vs. MET + SU + INS** | | |
| Russell-Jones 2009 | 18 - 80 years.  HbA_1c_ of 7.5 - 10.0% if on OAD monotherapy or 7.0 - 10.0% if on OAD combination therapy.  BMI ≤ 45 kg/m^2^. | - INS use with 3 months prior to screening.  - Impaired hepatic or renal function, clinically significant CVD, proliferative retinopathy or maculopathy, hypertension, cancer, pregnancy, recurrent hypoglycaemia or hypoglycaemia unawareness, hepatitis B, hepatitis C or use of mediations (other than OADs) that could affect blood glucose levels. |
| **MET + SU vs. MET + SU + SGLT2-i** | | |
| Wilding 2013 | 18 - 80 years.  HbA1c ≥ 7.0% to ≤ 10.5%.  On MET +SU. | Known complication of type 2 diabetes.  Requirement of insulin therapy.  Serum creatinine level higher than ULN.  Symptomatic urinary tract infection or genital infection.  Significant cardiovascular disease. |
| **MET + SU + TZD vs. MET + SU + INS** | | |
| Rosenstock 2006 | 18 - 70 years.  HbA_1c_ of 7.5 - 11.0%.  Drug naïve patients (no more than a short-term course of OADs (≤ 15 days) in the 12 weeks prior to screening).  Inadequate glycaemic control on diet and exercise alone. | Clinically significant renal, hepatic or haematological disease, uncontrolled hypertension, use of corticosteroids, CVD, use of an investigational agent within 30 days of the study, history of severe oedema, metabolic acidosis or diabetic ketoacidosis. |
| **MET + SU + GLP-1-RA vs. MET + SU + INS** | | |
| Bergenstal 2009 | - Duration of known T2DM > 6 months.  - 18 - 80 years.  - HbA_1c_ ≥ 8.0%.  - Treated with MET (> 1,500 mg/day) and a SU (> half the maximal dose) for 3 months prior to screening.  - INS naïve. | - Significant CVD, hepatic or renal insufficiency.  - Use of TZDs, α-glycosidase inhibitors or meglitinides within 6 months.  - History of an eating disorder or receiving treatment with a weight-reducing diet. |
| Heine 2005 | 30 - 75 years.  HbA_1c_ of 7.0 - 10.0%.  BMI of 25 - 45 kg/m^2^; Stable body weight (> 3 months).  - Treated with stable and maximally effective doses of MET and a SU for > 3 months before screening. | - Participation in an interventional medical, surgical or pharmaceutical study with previous 30 days.  - > 3 episodes of hypoglycaemia with 6 months prior to screening.  - Undergoing therapy for malignant disease (except basal/squamous cell carcinoma).  - CVD, serum creatinine concentration of > 135 µmol/L for men or > 110 µmol/L for women.  - Long-term use of systemic glucocorticoid therapy or use of a prescription medication to promote weight loss within previous 3 months.  - Treatment with INS (> 2 consecutive weeks), TZDs, α-glucosidase inhibitors or meglitinides within 3 months. |
| Nauck 2007 | 30 - 75 years.  HbA_1c_ of 7.0 - 11.0%.  BMI of 25 - 40 kg/m^2^; Stable body weight (> 3 months).  - Suboptimal glycaemic control despite receiving optimally effective doses of MET and an SU for > 3 months. | - > 3 episodes of hypoglycaemia in the 6 months prior to screening.  - Use of prescription drugs to promote weight loss within previous 3 months.  - Treated with INS, TZDs, α-glucosidase inhibitors or meglitinides for > 2 weeks within previous 3 months. |
| **MET + SU + SGLT2-i vs. MET + SU + DPP-4-i** | | |
| Schernthaner 2013 | ≥18 years old.  HbA1c 7.0 - 10.5%.  On combination therapy of MET+SU | Cardiovascular disease.  Treatment with either a PPARg agonist, or any anti-hyperglycaemic agents.  Renal imparement. |
| **MET + SU + TZD vs. MET + SU + DPP-4-i** | | |
| Liu 2013 | ≥18 years old.  HbA1c 7.0 - 11%.  On combination therapy of MET+SU | Insulin use within 12 weeks.  Contraindications for the use of pioglitazone or sitagliptin.  Impaired renal function.  Current or planned pregnancy, or lactation. |
| **MET + TZD vs. MET + TZD + DPP-4-i** | | |
| Bosi 2011 | - 18 - 80 years.  - Inadequate glycaemic control defined as:  (i) HbA_1c_ of 7.0 - 10.0% on MET (1,500mg or maximally tolerated dose) and PIO 30mg for > 2 months prior to screening; or  (ii) HbA_1c_ of 7.5% on MET and another OAD (excluding PIO or DPP-4-i) and subsequently a HbA_1c_ of 7.0 - 10.0% after switching and stabilization with MET and PIO 30 mg for 16 weeks.  - BMI of 23 - 45 kg/m^2^.  - FPG < 15.3 mmol/L.  - Fasting plasma C-peptide ≥ 0.26 nmol/L.  - < 7 days of antidiabetic therapy (other than metformin and pioglitazone) within 2 months of screening.  - BP of < 160/100 mmHg. | - CVD or any other severe disease. |
| DeFronzo 2012 | - 18 - 80 years.  - HbA_1c_ of 7.5 - 10.0% after stabilization period.  - BMI of 23 - 45 kg/m^2^.  - Inadequate glycaemic control despite MET monotherapy (>1,500 mg/day) for > 2 months.  - Fasting C-peptide ≥ 0.26 nmol/L  - BP of < 160/100 mmHg. | - Oral of systemic glucocorticoids or weight loss drugs within 3 months of screening.  - Urine albumin/creatinine ratio > 113 mg/mmol, history of laser treatment for proliferative retinopathy within 6 months, treated diabetic gastroparesis or CVD. |
| **MET + TZD + SU vs. MET + TZD + DPP-4-i** | | |
| DeRosa 2013 | - > 18 years.  - HbA_1c_ > 8.0%.  - BMI of 25 - 30 kg/m^2^.  - Naïve to treatment. | - History of ketoacidosis, rapidly progressive diabetic retinopathy, nephropathy or neuropathy, impaired hepatic or renal function, severe anaemia or severe CVD.  - Pregnancy or breastfeeding. |

BMI = body mass index; BP = blood pressure; CrCl = creatinine clearance; CVD = cardiovascular disease; DPP-4-i = dipeptidyl peptidase-4 inhibitors; FBG = fasting blood glucose; FPG = fasting plasma glucose; GIT = gastrointestinal tract; GLP-1-RA = glucagon-like peptide-1 receptor agonist; HbA_1c_ = glycated haemoglobin; INS = insulin; MET = metformin; MI = myocardial infarction; OAD = oral antidiabetic drug; PIO = pioglitazone; SU = sulfonylurea; T1DM = type 1 diabetes mellitus; T2DM = type 2 diabetes mellitus; TZD = thiazolidinedione; SGLT2-i = sodium glucose co-transporter 2 inhibitor.

## Baseline characteristics

### Table 4: **Key baseline characteristics from the triple therapy trials**

|  | **MET + SU vs.** | | | | | | | | | |
| --- | --- | --- | --- | --- | --- | --- | --- | --- | --- | --- |
|  | **MET + SU + DPP-4-i** | | | | | | | | | |
|  | **Hermansen 2007** | | | | **Owens 2011** | | **Lukashevich 2014** | | **Moses 2013** | |
|  | **SU + SIT** | **MET + SU**  **+ SIT** | **SU + PBO** | **MET + SU + PBO** | **MET + SU**  **+ LIN** | **MET + SU + PBO** | **MET+SU+VIL** | **MET + SU + PBO** | **MET+SU+SAX** | **MET + SU + PBO** |
| Trial duration | 6 months | | | | 6 months | | 6 months | | 6 months | |
| N | 441 | | | | 1,055 | | 318 | | 257 | |
| n | 106 | 116 | 106 | 113 | 792 | 263 | 158 | 160 | 129 | 128 |
| Male; n (%) | 56 (53%) | 61 (53%) | 58 (55%) | 59 (52%) | 371 (47%) | 127 (48%) | 80 (50.6%) | 72 (45%) | 80 (62.0%) | 74 (57.8%) |
| Race  Asian; n (%)  Black; n (%)  White; n (%)  Other; n (%) | 6 (6%)  7 (7%)  61 (58%)  32 (30%) | 16 (14%)  3 (3%)  75 (65%)  22 (19%) | 12 (11%)  3 (3%)  59 (56%)  32 (30%) | 13 (12%)  9 (8%)  81 (72%)  10 (9%) | 404 (51%)  6 (1%)  376 (47%)  6 (1%) | 141 (54%)  2 (1%)  116 (44%)  4 (2%) | 116 (73.4%)  34 (21.5%)  8 (5.1%) | 116 (72.5%)  38 (23.8%)  6 (3.8%) | 70 (54.3%)  59 (45.7%) | 71 (55.5%)  57 (44.5%) |
| Age, mean; years (SD) | 54.4 (10.3) | 56.6 (8.8) | 55.2 (10.2) | 57.7 (8.9) | 58.3 (9.9) | 57.6 (9.7) | 55.3 (10.2) | 55 (11.1) | 57.2 (9.6) | 56.8 (11.5) |
| Weight, mean; kg (SD) | 85.8 (22.5) | 87.2 (19.7) | 85.1 (22.6) | 86.7 (21.1) | 76.5 (16.8) | 76.8 (16.8) | 73.6 | 72.3 | 82.4 (19.9) | 80.3 (18.5) |
| BMI, mean; kg/m^2^ (SD) | 31.0 (6.7) | 31.3 (5.9) | 30.7 (6.4) | 30.7 (6.2) | 28.4 (4.8) | 28.2 (4.5) | 27.9 (4.6 ) | 28.0 (4.5) | 29.4 (5.3) | 29.1 (4.9) |
| Duration of diabetes, mean; years (SD) | 7.2 (5.0) | 9.3 (5.7) | 8.0 (6.5) | 10.6 (6.8) | - | - | 7.1 (6.2) | 7.5 (6.1) |  |  |
| HbA_1c_, mean; %, (SD) | 8.4% (0.8) | 8.3% (0.7) | 8.4% (0.8) | 8.3% (0.7) | 8.2% (0.0) | 8.1% (0.1) | 8.7% (0.9) | 8.8% (0.9) | 8.4% (0.9) | 8.2% (0.8) |

BMI = body mass index; DPP-4-i = dipeptidyl peptidase-4 inhibitors; HbA_1c_ = glycated haemoglobin; LIN = linagliptin; MET = metformin; PBO = placebo; SAX = saxagliptin; SD = standard deviation; SIT = sitagliptin; SU = sulfonylurea.

Table 4  **(cont)**: **Key baseline characteristics from the triple therapy trials**

|  | **MET + SU vs.** | | | | | | | | | | |
| --- | --- | --- | --- | --- | --- | --- | --- | --- | --- | --- | --- |
|  | **MET + SU + SGLT2-i** | | | **MET + SU + TZD** | | **MET + SU + GLP-1-RA** | | | **MET + SU + GLP-1-RA**  **vs. MET + SU + INS** | | |
|  | **Wilding 2013** | | | **Dailey 2004** | | **Kendall 2005** | | | **Russell-Jones 2009** | | |
|  | **MET + SU**  **+ PBO** | **MET+SU+** **CAN 100 mg** | **MET+SU+ CAN 300 mg** | **MET + SU**  **+ ROS** | **MET + SU**  **+ PBO** | **MET + SU + EXN 5** | **MET + SU + EXN 10** | **MET + SU + PBO** | **MET + SU**  **+ LIR** | **MET + SU + INS Gla** | **MET + SU**  **+ PBO** |
| Trial duration | 12 months | | | 6 months | | 7 months | | | 6 months | | |
| N | 469 | | | 365 | | 733 | | | 581 | | |
| n | 156 | 157 | 156 | 181 | 184 | 245 | 241 | 247 | 232 | 234 | 115 |
| Male; n (%) | 76 (48.7%) | 76 (48.4%) | 87 (55.8%) | 105 (58%) | 112 (61%) | 145 (59%) | 143 (59%) | 138 (56%) | 132 (57%) | 140 (60%) | 56 (49%) |
| Race  Asian; n (%)  Black; n (%)  White; n (%)  Other; n (%) | 2 (1.3%)  10 (6.4%)  128 (82.1%)  16 (10.3%) | 2 (1.3%)  5 (3.2%)  132 (84.1%)  18 (11.5%) | 0  11 (7.1%)  127 (81.4%)  18 (11.5%) | -  8 (4%)  139 (77%)  34 (19%) | -  20 (11%)  130 (71%)  34 (18%) | 7 (3%)  25 (10%)  169 (69%)  44 (18%) | 7 (3%)  28 (12%)  160 (66%)  46 (19%) | 4 (2%)  30 (12%)  167 (68%)  46 (19%) | -  -  -  - | -  -  -  - | -  -  -  - |
| Age, mean; years (SD) | 56.8 (8.3) | 57.4 (10.5) | 56.1 (8.9) | 57 (9) | 57 (10) | 55 (9) | 55 (10) | 56 (10) | 57.6 (9.5) | 57.5 (10.5) | 57.5 (9.6) |
| Weight, mean; kg (SD) | 91.2 (22.6) | 93.8 (22.6) | 93.5 (22.0) | 93 (18) | 93 (18) | 97 (19) | 98 (21) | 99 (18) | 85.5 (19.4) | 85.0 (17.9) | 85.7 (16.7) |
| BMI, mean; kg/m^2^ (SD) | 32.7 (6.8) | 33.3 (6.3) | 33.2 (6.3) | 32 (5) | 32 (5) | 33 (6) | 34 (6) | 34 (5) | 30.4 (5.3) | 30.3 (5.3) | 31.3 (5.0) |
| Duration of diabetes, mean; years (SD) | 10.3 (6.7) | 9.0 (5.7) | 9.4 (6.4) | 9 (7) | 9 (6) | 8.7 (5.9) | 8.7 (6.4) | 9.4 (6.2) | 9.2 (5.8) | 9.7 (6.4) | 9.4 (6.2) |
| HbA_1c_, mean; %, (SD) | 8.1 (0.9) | 8.1 (0.9) | 8.1 (0.9) | 8.1% (0.9) | 8.1% (0.8) | 8.5% (1.0) | 8.5% (1.1) | 8.5% (1.0) | 8.3% (0.9) | 8.2% (0.9) | 8.3% (0.9) |

BMI = body mass index; EXN = exenatide; Gla = glargine; GLP-1-RA = glucagon-like peptide-1 receptor agonist; HbA_1c_ = glycated haemoglobin; INS = insulin; LIR = liraglutide; MET = metformin; PBO = placebo; ROS = rosiglitazone; SD = standard deviation; SU = sulfonylurea; TZD = thiazolidinedione; CAN = Canagliflozin; SGLT2-i = sodium glucose co-transporter 2 inhibitor.

Table 4  **(cont): Key baseline characteristics from the triple therapy trials**

|  | **MET + SU + TZD vs.**  **MET +SU + INS** | | **MET + SU + GLP-1-RA vs. MET + SU + INS** | | | | | | |
| --- | --- | --- | --- | --- | --- | --- | --- | --- | --- |
|  | **Rosenstock 2006** | | **Bergenstal 2009** | | | **Heine 2005** | | **Nauck 2007** | |
|  | **MET + SU**  **+ ROS** | **MET + SU**  **+ INS Gla** | **MET + SU**  **+ EXN** | **MET + SU**  **+ INS (od)** | **MET + SU**  **+ INS (bid)** | **MET + SU**  **+ EXN** | **MET + SU**  **+ INS** | **MET + SU**  **+ EXN** | **MET + SU**  **+ INS** |
| Trial duration | 6 months | | 6 months | | | 6 months | | 12 months | |
| N | 216 | | 372 | | | 549 | | 501 | |
| n | 112 | 104 | 124 | 124 | 124 | 282 | 267 | 248 | 253 |
| Male; n (%) | 65 (58%) | 47 (45%) | 60 (48%) | 60 (48%) | 59 (48%) | 155 (55%) | 151 (57%) | 122 (49%) | 134 (53%) |
| Race  Asian; n (%)  Black; n (%)  White; n (%)  Other; n (%) | -  -  -  - | -  -  -  - | 2 (2%)  24 (19%)  79 (64%)  19 (15%) | 3 (2%)  23 (19%)  84 (68%)  14 (11%) | 2 (2%)  33 (27%)  74 (60%)  15 (12%) | 5 (2%)  2 (1%)  225 (80%)  50 (18%) | 2 (1%)  3 (1%)  215 (81%)  47 (18%) | -  -  -  - | -  -  -  - |
| Age, mean; years (SD) | 55.3 (11.4) | 55.9 (10.5) | 52.5 (10.6) | 51.8 (10.9) | 53.4 (10.0) | 59.8 (8.8) | 58.0 (9.5) | 59 (9) | 58 (9) |
| Weight, mean; kg (SD) | - | - | 96.6 (24.0) | 96.9 (25.0) | 93.8 (24.0) | 87.5 (16.9) | 88.3 (17.9) | 83.4 (15.6) | 85.5 (15.7) |
| BMI, mean; kg/m^2^ (SD) | 33.6 (6.3) | 34.6 (7.0) | 34.2 (7.1) | 33.7 (7.1) | 33.5 (7.4) | 31.4 (4.4) | 31.3 (4.6) | 30.2 (4.2) | 30.6 (4.0) |
| Duration of diabetes, mean; years (SD) | 8.1 (5.1) | 8.5 (5.8) | 8.6 (5.9) | 8.4 (6.3) | 9.9 (5.6) | 9.9 (6.0) | 9.2 (5.7) | 10.0 (6.2) | 9.8 (6.3) |
| HbA_1c_, mean; % (SD) | 8.7% (1.0) | 8.8% (1.0) | 10.2% (1.5) | 10.1% (1.8) | 10.3% (1.9) | 8.2% (1.0) | 8.3% (1.0) | 8.6% (1.1) | 8.6% (1.0) |

BMI = body mass index; EXN = exenatide; GLP-1-RA = glucagon-like peptide-1 receptor agonist; HbA_1c_ = glycated haemoglobin; INS = insulin; MET = metformin; od = once daily; bid = twice daily; SD = standard deviation; ROS = rosiglitazone; SU = sulfonylurea; TZD = thiazolidinedione

Table 4  **(cont): Key baseline characteristics from the triple therapy trials**

|  | **MET + TZD vs. MET + TZD + DPP-4-i** | | | | | | | | | | | | | |
| --- | --- | --- | --- | --- | --- | --- | --- | --- | --- | --- | --- | --- | --- | --- |
|  | **Bosi 2011** | | **DeFronzo 2012** | | | | | | | | | | | |
|  | **MET + PIO 30 + ALO 25** | **MET + PIO 45** | **MET + PBO** | **MET + ALO 12.5** | **MET +**  **ALO 25** | **MET +**  **PIO 15** | **MET +**  **PIO 15 +**  **ALO 12.5** | **MET +**  **PIO 15 +**  **ALO 25** | **MET +**  **PIO 30** | **MET+**  **PIO 30 +**  **ALO 12.5** | **MET +**  **PIO 30 +**  **ALO 25** | **MET +**  **PIO 45** | **MET +**  **PIO 45 +**  **ALO 12.5** | **MET +**  **PIO 45 + ALO 25** |
| Trial duration | 12 months | | 6 months | | | | | | | | | | | |
| N | 803 | | 1,554 | | | | | | | | | | | |
| n | 404 | 399 | 129 | 128 | 129 | 130 | 130 | 130 | 129 | 130 | 130 | 129 | 130 | 130 |
| Male; n (%) | 210 (52%) | 204 (51%) | 61 (47%) | 67 (52%) | 50 (39%) | 61 (47%) | 60 (46%) | 61 (47%) | 63 (49%) | 54 (42%) | 55 (42%) | 53 (41%) | 60 (46%) | 52 (40%) |
| Race  Asian; n (%)  Black; n (%)  White; n (%)  Other; n (%) | 79 (20%)  41 (10%)  245 (61%)  42 (10%) | 78 (20%)  36 (9%)  256 (64%)  29 (7%) | 5 (4%)  8 (6%)  93 (72%)  23 (18%) | 14 (11%)  6 (5%)  89 (70%)  19 (15%) | 15 (12%)  5 (4%)  80 (62%)  29 (22%) | 11 (8%)  8 (6%)  85 (65%)  26 (20%) | 9 (7%)  4 (3%)  95 (73%)  22 (17%) | 7 (5%)  3 (2%)  96 (74%)  24 (18%) | 10 (8%)  6 (5%)  96 (74%)  17 (13%) | 5 (4%)  2 (2%)  107 (82%)  16 (12%) | 12 (9%)  5 (4%)  85 (65%)  28 (21%) | 12 (9%)  9 (7%)  85 (66%)  23 (18%) | 8 (6%)  9 (7%)  92 (71%)  21 (16%) | 12 (9%)  3 (2%)  93 (72%)  22 (17%) |
| Age, mean; years (SD) | 54.3 (9.9) | 55.9 (9.9) | 55.2 (9.9) | 53.1 (9.6) | 53.7 (9.3) | 54.1 (9.5) | 53.6 (9.9) | 54.9 (9.2) | 56.1 (9.4) | 55.0 (9.1) | 54.4 (9.7) | 54.5 (9.7) | 54.0 (9.8) | 54.2 (8.9) |
| Weight, mean; kg (SD) | 88.2 (18.9) | 88.0 (19.3) | - | - | - | - | - | - | - | - | - | - | - | - |
| BMI, mean; kg/m^2^ (SD) | 31.5 (5.3) | 31.6 (5.2) | 30.6 (4.8) | 31.0 (5.1) | 31.5 (5.7) | 31.3 (5.3) | 31.5 (5.0) | 30.8 (4.7) | 31.4 (5.4) | 31.1 (5.1) | 31.9 (5.6) | 30.7 (4.7) | 31.5 (5.2) | 30.6 (4.8) |
| Duration of diabetes, mean; years (SD) | 7.5 (5.2) | 6.9 (4.6) | 6.0 (5.0) | 6.2 (5.6) | 5.6 (4.9) | 5.7 (4.8) | 6.1 (5.5) | 6.9 (5.5) | 7.6 (7.1) | 5.8 (5.1) | 6.6 (6.0) | 5.7 (4.2) | 6.6 (5.3) | 6.2 (5.0) |
| HbA_1c_, mean; % (SD) | 8.3% (0.8) | 8.1% (0.8) | 8.5% (0.6) | 8.6% (0.7) | 8.6% (0.7) | 8.5% (0.7) | 8.5% (0.7) | 8.5% (0.7) | 8.5% (0.7) | 8.5% (0.7) | 8.5% (0.7) | 8.5% (0.7) | 8.5% (0.7) | 8.6% (0.7) |

ALO = alogliptin; BMI = body mass index; DPP-4-i = dipeptidyl peptidase-4 inhibitors; HbA_1c_ = glycated haemoglobin; INS = insulin; MET = metformin; PBO = placebo; PIO = pioglitazone; SD = standard deviation; TZD = thiazolidinedione

Table 4  **(cont): Key baseline characteristics from the triple therapy trials**

|  | **MET + SU + DPP-4-i vs.** | | | | **MET + TZD + SU + vs.** | |
| --- | --- | --- | --- | --- | --- | --- |
|  | **MET + SU + SGLT2-i** | | **MET + SU + TZD** | | **MET + TZD + DPP-4-i** | |
|  | **Schernthaner 2013** | | **Liu 2013** | | **DeRosa 2013** | |
|  | **MET + SU + SIT** | **MET + SU + CAN** | **MET + SU + PIO** | **MET + SU + SIT** | **MET + PIO + SU** | **MET + PIO + SIT** |
| Trial duration | 12 months | | 6 months | | 12 months | |
| N | 755 | | 120 | | 453 | |
| n | 378 | 377 | 60 | 60 | 225 | 228 |
| Male; n (%) | 215 (56.9) | 207 (54.9) | 23 (38.3%) | 22 (36.7%) | 114 (51%) | 113 (50%) |
| Race  Asian; n (%)  Black; n (%)  White; n (%)  Other; n (%) | 65 (17.2)  45 (11.9)  240 (63.5)  28 (7.4) | 67 (17.8)  43 (11.4)  245 (65.0)  22 (5.8) | - | - | -  -  -  - | -  -  -  - |
| Age, mean; years (SD) | 56.7 (9.3%) | 56.6 (9.6%) | 58.1 (8.3) | 60.1 (8.9) | - | - |
| Weight, mean; kg (SD) | 89.1 (23.2) | 87.4 (23.2) | 65.4 (10.4) | 69.4 (13.6) | 78.4 (7.5) | 78.9 (7.9) |
| BMI, mean; kg/m^2^ (SD) | 31.7 (6.9) | 31.5 (6.9) | 25.7 (3.7) | 26.6 (4.6) | 27.3 (2.1) | 27.6 (2.4) |
| Duration of diabetes, mean; years (SD) | 9.7 (6.3) | 9.4 (6.1) | 7.8 (3.9) | 7.8 (4.3) | - | - |
| HbA_1c_, mean; % (SD) | 8.1 (0.9) | 8.1 (0.9) | 8.54 (0.97) | 8.27 (0.86) | 7.3 (0.8) | 7.1 (0.7) |

BMI = body mass index; DPP-4-i = dipeptidyl peptidase-4 inhibitor; GLP-1-RA = glucagon-like peptide-1 receptor agonist; HbA_1c_ = glycated haemoglobin;; MET = metformin; PBO = placebo; PIO = pioglitazone; CAN = Canagliflozin; SD = standard deviation; SIT = sitagliptin; SU = sulfonylurea; TZD = thiazolidinedione; SGLT2-i = sodium glucose co-transporter 2 inhibitor.

## Results of Efficacy and adverse events in included trials

### Table 5: Results of the triple therapy trials

| **MET + SU vs.** | **MET + SU + DPP-4-i** | | | | | | | | | |
| --- | --- | --- | --- | --- | --- | --- | --- | --- | --- | --- |
| **Publication** | **Hermansen 2007** | | | | **Owens 2011** | | **Lukashevich 2014** | | **Moses 2013** | |
|  | **MET + SU**  **+ SIT** | **MET + SU**  **+ PBO** | **SU + SIT** | **SU + PBO** | **MET + SU**  **+ LIN** | **MET + SU + PBO** | **MET + SU + VIL** | **MET + SU + PBO** | **MET + SU + SAX** | **MET + SU + PBO** |
| Trial duration | 6 months | | | | 6 months | | 6 months | | 6 months | |
| n | 116 | 113 | 106 | 106 | 792 | 263 | 158 | 160 | 129 | 128 |
| HbA_1c_*; %  (95% CI or SD) | -0.59  (-0.44, -0.74) | 0.3  (0.14, 0.45) | -0.3  (-0.12, -0.48) | 0.27  (0.09, 0.45) | -0.72  (0.03) | -0.10  (0.05) | −1.01 | 0.25 | -0.74  (-0.89, -0.60) | -0.08  (-0.23, 0.07) |
| BW*; kg  (95% CI or SD) | 0.4  (-0.1, 0.9) | -0.7  (-0.1, -1.4) | 1.1  (0.5, 1.8) | 0.0  (-0.6, 0.7) | 0.27  (0.09) | -0.06  (0.16) | 0.5 | -0.1 | 0.2 (2.2) | -0.6 (2.1) |
| Any AE; % | 62.9% | 53.1% | 55.7% | 40.6% | 66.3% | 59.7% | 50.0% | 47.5% | 62.8% | 71.1% |
| SAE; % | 6.0% | 1.8% | 4.7% | 5.7% | 3.2% | 3.8% | 1.9% | 1.3% | 2.3% | 5.5% |
| Hypo-G; % | 16.4% | 0.9% | 7.5% | 2.8% | 22.7% | 14.8% | 5.1% | 1.8% | 10.1% | 6.3% |

| **MET + SU vs.** | **MET + SU + TZD** | | **MET + SU + GLP-1-RAs** | | | **MET + SU + GLP-1-RAs**  **vs. MET + SU + INS** | | | **MET + SU vs. MET + SU + SGLT2-i** | | |
| --- | --- | --- | --- | --- | --- | --- | --- | --- | --- | --- | --- |
| **Publication** | **Dailey 2004** | | **Kendall 2005** | | | **Russell-Jones 2009** | | | **Wilding 2013** | | |
| **Triple therapy** | **MET + SU + ROS** | **MET + SU + PBO** | **MET + SU + EXN 5** | **MET + SU + EXN 10** | **MET + SU + PBO** | **MET + SU + LIR** | **MET + SU + INS Gla** | **MET + SU + PBO** | **MET + SU**  **+ PBO** | **MET+SU+** **CAN 100 mg** | **MET+SU+ CAN 300 mg** |
| Trial duration | 6 months | | 7 months | | | 6 months | | | 12 months | 12 months | 12 months |
| n | 181 | 184 | 245 | 241 | 247 | 232 | 234 | 115 | 156 | 157 | 156 |
| HbA_1c_*; % (SD) | -0.9 | 0.1 | -0.55 (0.07) | -0.77 (0.08) | 0.23  (0.07) | -1.33 | -1.09 | -0.24 | –0.13% (0.08) | –0.85% (0.08) | –1.06% (0.06) |
| BW*; kg (SD) | 3.0 | 0.03 | -1.6  (0.2) | -1.6  (0.2) | -0.9  (0.2) | -1.8  (0.33) | 1.6  (0.33) | -0.42  (0.39) | -0.8 (0.25) | -1.9 (0.25) | -2.5 (0.25) |
| Any AE; % | - | - | - | - | - | - | - | - | - | - | - |
| SAE; % | 1.7% | 4.3% | 6.1% | 6.2% | 8.1% | 3.9% | 6.8% | 7.0% | - | - | - |
| Hypo-G; % | 52.5% | 24.5% | 19.2% | 27.8% | 12.6% | 19.8% | 29.1% | 16.5% | 8.4% | 22.0% | 26.5% |

* mean change from baseline

AE = adverse event; BW = body weight; CI = confidence interval; DPP-4-i = dipeptidyl peptidase-4 inhibitor; EXN = exenatide; Gla = glargine; GLP-1-RA = glucagon-like peptide-1 receptor agonist; HbA_1c_ = glycated haemoglobin; Hypo-G = hypoglycaemia; INS = insulin; LIN = linagliptin; LIR = liraglutide; MET = metformin; PBO = placebo; ROS = rosiglitazone; SAE = serious adverse event; SD = standard deviation; SIT = sitagliptin; SU = sulfonylurea; TZD = thiazolidinedione; CAN = Canagliflozin; SGLT2-i = sodium glucose co-transporter 2 inhibitor.

Table 5 **(cont)**: Results of the triple therapy trials

|  | **MET + SU + TZD vs.**  **MET + SU + INS** | | | **MET + SU + GLP-1-RA vs. MET + SU + INS** | | | | | | | | | | | | | **MET + TZD + SU vs.**  **MET + TZD + DPP-4-i** | | |
| --- | --- | --- | --- | --- | --- | --- | --- | --- | --- | --- | --- | --- | --- | --- | --- | --- | --- | --- | --- |
| **Publication** | **Rosenstock 2006** | | | **Bergenstal 2009** | | | | | | **DeRosa 2013** | | | **Nauck 2007** | | | | **DeRosa 2013** | | |
| **Triple therapy** | **MET + SU**  **+ ROS** | **MET + SU**  **+ INS Gla** | | **MET + PIO + SU** | | **MET + PIO + SIT** | | **MET + SU**  **+ INS (bid)** | | **MET + SU**  **+ EXN** | | **MET + SU**  **+ INS** | **MET + SU**  **+ EXN** | | **MET + SU**  **+ INS** | | **MET + PIO + SU** | | **MET + PIO + SIT** |
| Trial duration | 6 months | | | 6 months | | | | | | 12 months | | | 12 months | | | | 12 months | | |
| n | 112 | 104 | | 225 | | 228 | | 124 | | 282 | | 267 | 248 | | 253 | | 225 | | 228 |
| HbA_1c_*; % (SD) | -1.51 | -1.66 | | -1.1  (0.5) | | -0.7  (0.2) | | -2.76 (1.79) | | -1.11 | | -1.11 | -1.04 (0.07) | | -0.89 (0.06) | | -1.1  (0.5) | | -0.7  (0.2) |
| BW*; kg (SD) | 3 (0.4) | 1.7 (0.4) | | 4.5  (1.6) | | -2.5  (0.9) | | 4.1 (5.4) | | -2.3 | | 1.8 | -2.5 (0.2) | | 2.9 (0.2) | | 4.5  (1.6) | | -2.5  (0.9) |
| Any AE; % | - | - | | - | | - | | - | | - | | - | 72.2% | | 48.6% | | - | | - |
| SAE; % | 9.8% | 4.8% | | - | | - | | - | | - | | - | 7.7% | | 4.3% | | - | | - |
| Hypo-G; % | 42.0% | 54.8% | | - | | - | | 61.3% | | - | | - | - | | - | | - | | - |
|  | **MET + TZD vs. MET + TZD + DPP-4-i** | | | | | | | | | | **MET + SU + SGLT2-i vs. MET + SU + DPP-4-i** | | | | | **MET + SU + TZD vs. MET + SU + DPP-4-i** | | | |
| **Publication** | **Bosi 2011** | | | | **DeFronzo 2012** | | | | | | **Schernthaner 2013** | | | | | **Liu 2013** | | | |
| **Triple therapy** | **MET + PIO 30 + ALO 25** | | **MET + PIO 45** | | **MET + PIO** | | **MET + PIO + ALO 12.5** | | **MET + PIO + ALO 25** | | **MET + SU + SIT** | | | **MET + SU + CAN** | | **MET + SU + PIO** | | **MET + SU + SIT** | |
| Trial duration | 12 months | | | | 6 months | | | | | | 12 months | | | | | 6 months | | | |
| n | 404 | | 399 | | 388 | | 390 | | 390 | | 378 | | | 377 | | 60 | | 60 | |
| HbA_1c_*; % (SD) | -0.7 | | -0.23 | | -0.9  (0.05) | | -1.4  (0.05) | | -1.4  (0.05) | | -0.92 (0.55) | | | -1.15 (0.55) | | -0.94 (0.21) | | -0.71 (0.21) | |
| BW*; kg (SD) | 1.1  (0.19) | | 1.6  (0.19) | | 1.5  (0.2) | | 1.8  (0.2) | | 1.9  (0.2) | | 0.3 (0.2) | | | -2.7 (0.2) | | 1.34 (0.32) | | -0.26 (0.32) | |
| Any AE; % | 71.5% | | 68.9% | | 60.8% | | 61.0% | | 64.4% | | - | | | - | | 51.7% | | 43.3% | |
| SAE; % | 5.0% | | 5.0% | | 3.4% | | 1.8% | | 3.1% | | - | | | - | | 0 | | 0 | |
| Hypo-G; % | 4.5% | | 1.5% | | 2.1% | | 1.0% | | 1.5% | | - | | | - | | 8.5 | | 10 | |

* mean change from baseline

AE = adverse event; BW = body weight; ALO = alogliptin; DPP-4-i = dipeptidyl peptidase-4 inhibitor; Gla = glargine; GLP-1-RA = glucagon-like peptide-1 receptor agonist; HbA_1c_ = glycated haemoglobin; Hypo-G = hypoglycaemia; INS = insulin; MET = metformin; PIO = pioglitazone; ROS = rosiglitazone; SAE = serious adverse event; SD = standard deviation; SIT = sitagliptin; SU = sulfonylurea; TZD = thiazolidinedione; CAN = Canagliflozin; SGLT2-i = sodium glucose co-transporter 2 inhibitor.
