# Supplementary material for: Triple therapy in type 2 diabetes; a systematic review and network meta-analysis
Source: PeerJ. 2015 Dec 7;3:e1461. doi: 10.7717/peerj.1461 (PMC4675096; doi:10.7717/peerj.1461)
Supplement: Supplemental Information 4 [file peerj-03-1461-s004.docx]

1. The rationale for conducting the meta-analysis;

With the wide range of medications for type 2 diabetes, identifying the right combination of triple therapy is difficult and problematic i.e; which combination provides better HbA1c control or helps weight reduction, etc. This systematic review and network analysis compares numerous triple therapy combinations to help fill this gap and aid professionals for decision making in patient care.

2. The contribution that the meta-analysis makes to knowledge in light of previously published related reports, including other meta-analyses and systematic reviews.

The systematic review is available to help policy makers identify the best treatment available and the problems associated with each of the combinations; information that was not previously available. The systematic review, not only provides information on which drug is comparatively more affective at controlling diabetes but also identifies other outcomes, including body weight and hypoglycaemia events, this can guide the clinician in tailoring the treatment for the individual patient’s needs. Another similar systematic review exists (Mearns ES et al. 2015); however, Mearns ES et al. (2015) was limited in its searches and only identified therapies that included metformin and sulphonylureas as the basic combination. Given that there are other dual therapy combinations available, our systematic searches were much more extensive and included other forms of therapy combinations, hence adding more depth to the analysis and enabling a richer interpretation of the current data.
